# Outstanding intraindividual genetic diversity in fissiparous planarians (*Dugesia*, Platyhelminthes) with facultative sex

**DOI:** 10.1186/s12862-019-1440-1

**Published:** 2019-06-20

**Authors:** Laia Leria, Miquel Vila-Farré, Eduard Solà, Marta Riutort

**Affiliations:** 1 Department de Genètica, Microbiologia i Estadística, Facultat de Biologia, Universitat de Barcelona, and Institut de Recerca de la Biodiversitat (IRBio), Barcelona, Catalonia Spain; 2 Max Planck Institute of Molecular Cell Biology and Genetics, Dresden, Germany

**Keywords:** Facultative sex, Fissiparous reproduction, Meselson effect, Mosaicism, Muller’s ratchet, Multilevel selection

## Abstract

**Background:**

Predicted genetic consequences of asexuality include high intraindividual genetic diversity (i.e., the Meselson effect) and accumulation of deleterious mutations (i.e., Muller’s Ratchet), among others. These consequences have been largely studied in parthenogenetic organisms, but studies on fissiparous species are scarce. Differing from parthenogens, fissiparous organisms inherit part of the soma of the progenitor, including somatic mutations. Thus, in the long term, fissiparous reproduction may also result in genetic mosaicism, besides the presence of the Meselson effect and Muller’s Ratchet. Dugesiidae planarians show outstanding regeneration capabilities, allowing them to naturally reproduce by fission, either strictly or combined with sex (facultative). Therefore, they are an ideal model to analyze the genetic footprint of fissiparous reproduction, both when it is alternated with sex and when it is the only mode of reproduction.

**Results:**

In the present study, we generate and analyze intraindividual cloned data of a nuclear and a mitochondrial gene of sexual, fissiparous and facultative wild populations of the species *Dugesia subtentaculata*. We find that most individuals, independently of their reproductive strategy, are mosaics. However, the intraindividual haplotype and nucleotide diversity of fissiparous and facultative individuals is significantly higher than in sexual individuals, with no signs of Muller’s Ratchet. Finally, we also find that this high intraindividual genetic diversity of fissiparous and facultative individuals is composed by different combinations of ancestral and derived haplotypes of the species.

**Conclusions:**

The intraindividual analyses of genetic diversity point out that fissiparous reproduction leaves a very special genetic footprint in individuals, characterized by mosaicism combined with the Meselson effect (named in the present study as the *mosaic Meselson effect*). Interestingly, the different intraindividual combinations of ancestral and derivate genetic diversity indicate that haplotypes generated during periods of fissiparous reproduction can be also transmitted to the progeny through sexual events, resulting in offspring showing a wide range of genetic diversity and putatively allowing purifying selection to act at both intraindividual and individual level. Further investigations, using *Dugesia* planarians as model organisms, would be of great value to delve into this new model of genetic evolution by the combination of fission and sex.

**Electronic supplementary material:**

The online version of this article (10.1186/s12862-019-1440-1) contains supplementary material, which is available to authorized users.

## Background

The fitness of an individual and its lineage largely depends on the number and viability of the offspring produced during its lifetime. In turn, the genetic background of offspring has a major role in their survival and adaptation, for example, when facing population bottlenecks or in the face of environmental changes [1, 2]. Thus, the reproductive strategy and how it shapes the genetic background of the offspring represents a key life history trait to understand how lineages survive in the wild and why some populations are maintained while others become extinct.

Sexual reproduction can generate new allelic combinations in the populations through recombination and outcrossing, which can be either favored by selection or selected against [3, 4]. This can potentially accelerate the evolutionary processes, promoting the genetic diversification of the populations [5, 6]. Asexual reproduction, on the other hand, is characterized by the production of descendants that are genetically highly similar to their progenitor, due to the absence of recombination and outcrossing. For this reason, at first, it was assumed that asexual species would show low levels of genetic diversity, both at the intraindividual level (heterozygosity) and between different individuals (from the same or from different populations). Nevertheless, unsuspected genetic variation at these two levels has been found in different asexual taxa. On the one hand, genetic variation between different individuals (within and between populations) has been attributed either to their recurrent origin from sexual lineages or to demographic expansions [7, 8]. On the other hand, genetic diversity of asexual species at the intraindividual level has been attributed to hybridization processes [9] or to the independent accumulation of mutations in the homologous alleles over generations in the absence of recombination and out-crossing (i.e., Meselson effect) [10–12]. Moreover, it has been proposed that long-lasting asexuality can promote an increased number of slightly deleterious mutations as a consequence of relaxed selection (the physical linkage among loci hinders selection’s ability to act upon loci independently), which in the long term can cause detrimental effects on the populations (i.e., Muller’s ratchet) [13–17]. However, most of these studies have been performed in parthenogenetic asexual organisms, while clonal reproduction by some type of fissioning is rarely considered, although this type of reproduction is known to exist in most phyla within metazoans [18].

Differing from sexual and parthenogenetic individuals, a zygotic bottleneck is absent in fissiparous organisms, and descendants inherit part of the soma of the progenitor, including somatic mutations. This adds a level of complexity since, in the long term, fissiparous individuals are predicted to show high levels of genetic mosaicism [19, 20], in addition to the possible occurrence of the Meselson effect and Muller’s ratchet. Mosaicism associated with clonal reproduction has long been known to occur in plants [21], but its existence in fissiparous metazoans has only been demonstrated in colonial corals at the intracolonial level [22]. Therefore, we not only miss a confirmed example of mosaicism in noncolonial fissiparous metazoans in natural conditions but also its characterization regarding the possible occurrence of the Meselson effect and Muller’s ratchet.

**Fig. 1:**
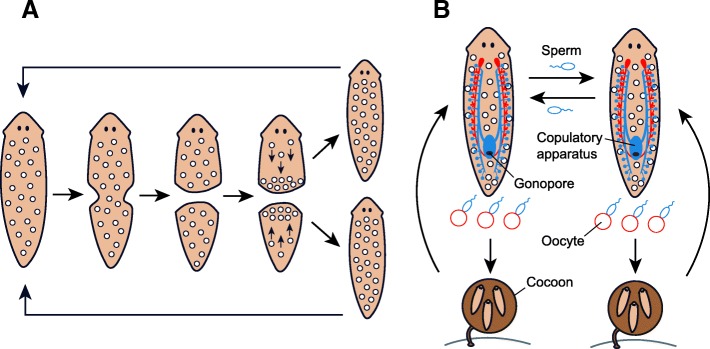
Schematic representation of fissiparous (**a**) and sexual (**b**) reproduction in planarians. **a** Fissiparous individuals do not develop the reproductive system and instead they reproduce by binary fission. After the fission process, the planarian stem cells or neoblasts (white dots) are recruited to the wound and regenerate the missing part of the individual through proliferation and differentiation. **b** Sexual individuals are hermaphrodites that show the full set of male (blue) and female (red) reproductive organs. In this case, the differentiated germ cells located in the ovaries and in the testes are the responsible for gamete production, while neoblasts of sexual individuals (white dots) do not participate in the reproductive process. After the cross-fertilization by the exchange of sperm, several embryos are encapsulated into a cocoon, which will hatch after few weeks from the oviposition

Planarians of the family Dugesiidae (Tricladida, Platyhelminthes) show outstanding regeneration capabilities among the metazoans [23]. Species such as *Schmidtea mediterranea* or several *Dugesia* species are indeed masters of regeneration [24, 25]. The only stem cells in the adult planarians are the neoblasts, distributed throughout most of their parenchyma (i.e., the connective tissue that fills the space between organs) and representing ∼25–30% of all planarian cells [26, 27]. Neoblasts are the only cells that divide mitotically and hence are responsible for all the cell and tissue renewal during regeneration and homeostasis [28, 29]. These extraordinary regeneration capabilities of planarians, due to neoblast activity, allow some species or some populations within a species to naturally reproduce by fission. Fissiparous individuals do not develop a reproductive system. Instead, they produce new individuals by performing a binary fission and subsequently regenerating the missing body parts (Fig. 1a). Therefore, fissiparous individuals need to rebuild all the lost structures and regain the original body proportions during each reproductive event. This process implies extensive body remodeling and neoblast migration and proliferation that, together with the animal’s longevity (they are theoretically immortal), opens the opportunity to amplify mutated neoblasts.

Therefore, besides being usually sexual, planarians can also reproduce asexually either by fission or by parthenogenesis, resulting in a group with an astonishing diversity of reproductive modes. Sexual individuals are simultaneous hermaphrodites (i.e., each individual possesses the entire set of male and female reproductive organs) (Fig. 1b). In general, sexual individuals are diploid and perform gametogenesis through normal meiosis from differentiated germ cells, which are in the ovaries and in the testes. Sexual individuals exhibit mutual insemination during copula and after fertilization, fertilized eggs and yolk cells are encapsulated into a cocoon, which is expelled through the gonopore and, a few weeks later, results in a variable number of juveniles hatching (Fig. 1b) [30]. Parthenogenetic individuals, on the other hand, are simultaneous hermaphrodites that need sperm to trigger the development of the zygote, without contributing its genetic content [31]. In general, asexual reproduction in planarians (either by fissiparity or by parthenogenesis) is linked to polyploidy and to chromosomal rearrangements [31]. Interestingly, these reproductive modes can operate either in different species, in different populations of the same species, or even in the same individual (facultative reproduction).

Facultative reproduction in the genus *Schmidtea* involves the alternation of parthenogenesis and sexual reproduction [32], while facultative individuals of *Dugesia* alternate fissiparity with sex ([33], and references therein). It could be thought that triploid facultative *Dugesia* individuals may in fact alternate fission with parthenogenesis, due to the disadvantages of polyploids during meiotic processes [34]. However, it has been demonstrated that triploid facultative *Dugesia* individuals can reproduce truly sexually through a special meiotic system [35]. These triploid facultative individuals are able to produce recombinant haploid sperm and recombinant diploid and haploid oocytes. Importantly, it has been shown that fissiparous planarians do not have a differentiated germline and thus, during the process of sexualization, the germline needs to be newly differentiated from neoblasts [36–38]. Therefore, in facultative *Dugesia*, somatic genetic diversity generated during periods of fissiparous reproduction could putatively be transmitted to descendants through sex.

An example of a species showing the whole variety of reproductive strategies (sexual, fissiparous and facultative) is *Dugesia subtentaculata*. At first, only strictly sexual (diploid, 2n = 16) and strictly fissiparous populations (triploid, 3n = 24) were known [39–41]. However, an extensive sampling across all its distributional range has resulted in the detection of not only more sexual and fissiparous populations, but also in many populations showing both sexual and fissiparous individuals [42]. A priori, these populations could be either a mix of strictly sexual and strictly fissiparous individuals or, could be constituted by facultative individuals (individuals that alternate between both types of reproduction). Whether they represent one or the other case can be genetically tested, in the first case we will expect to find two independent lineages in the populations, while in the second only a genetic lineage will be found. This species is therefore a potentially ideal model to analyze the genetic footprint that fissiparous reproduction leaves in organisms, and potentially also when it is combined with sex (provided that our genetic analyses demonstrate that the mixed populations bear a single genetic lineage and hence are facultative).

Here, we analyze the intraindividual genetic diversity, by cloning PCR products of two molecular markers (one mitochondrial and one nuclear), of individuals coming from a total of 10 natural populations of *D. subtentaculata* showing either sexual, fissiparous or putative-facultative reproductive strategies, to investigate the following predictions under an evolutionary framework: (1) the existence of high levels of mosaicism in purely fissiparous individuals as well as the absence of mosaicism in purely sexual individuals, (2) the existence of the Meselson effect and Muller’s ratchet in fissiparous individuals and their absence in purely sexual ones, and (3) if putative-facultative populations prove to be facultative, we would expect to find in their individuals a characteristic genetic pattern different from that in exclusively sexual or fissiparous populations.

Our results demonstrate in the first place that individuals from the populations bearing sexual and fissiparous individuals are facultative. Moreover, the data obtained provide evidence for the existence of mosaicism in freshwater planarians accompanied by the Meselson effect in fissiparous and facultative individuals, but with no Muller’s ratchet. Additionally, our results point out that the combination of fissiparous reproduction with occasional sex results in an efficient way of generating high levels of genetic diversity controlled by selection that may act at two different levels (intraindividual and individual), which represents an entirely novel model of genetic evolution among metazoans.

## Methods

### Sampling

**Fig. 2:**
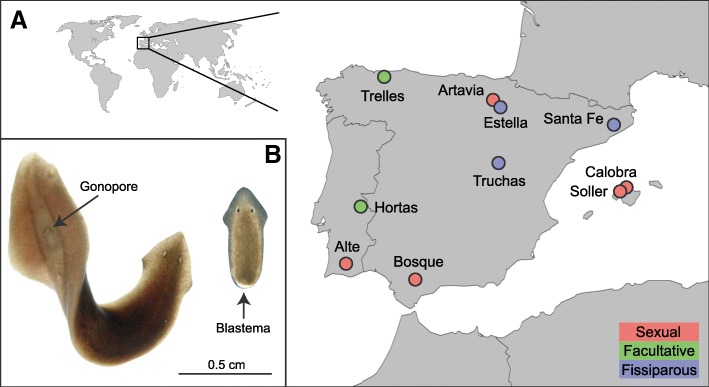
Distribution map of the samples used in this study (**a**) and appearance of an individual reproducing sexually (left) and by fission (right) (**b**). For each locality, its code name and the reproductive strategy of the population are indicated

We studied a total of 10 natural populations of *D. subtentaculata*, 5 sexual, 2 facultative and 3 fissiparous, covering almost the maximum area of distribution of the species, which includes Southern France, the Iberian Peninsula, Mallorca (Balearic Islands) and Northern Africa (Fig. 2a; see Additional file 1: Table S1). An average of 15 individuals per population were collected and observed under the stereomicroscope under field conditions or shortly after. Sexual individuals were identified by the presence of a gonopore (external aperture of the copulatory apparatus) (indicated by an S in each individual code) and fissiparous individuals by the occurrence of a blastema (regenerating part of tissue after a process of fission) (indicated by an A in each individual code) (Fig. 2b). The individuals with neither blastema nor gonopore have no assigned identification letter. The populations were classified as sexual if most individuals presented a gonopore and none a blastema, facultative when both individuals with a gonopore and individuals with a blastema were detected, and fissiparous when individuals with a blastema were detected and none had a gonopore. Subsequently, three individuals per population (five in Hortas) were fixed in 100% ethanol for the genetic analysis, while three other individuals were kept alive for the ploidy analysis. In facultative populations, at least one sexual and one fissiparous individual were included in the analyses.

### Ploidy identification

The ploidy level of the populations of Calobra, Soller and Santa Fe was extracted from the literature [40], while the ploidy level of the population Bosque was inferred by karyotyping in a companion study [42]. The ploidy level of the rest of the populations was determined by flow cytometry. Since our protocol for flow cytometry requires a high quantity of tissue (Additional file 2: Figure S1) only big sized animals could be used jointly for the ploidy identification and genetic analysis (populations of Alte, Artavia and Hortas). In the rest of populations different individuals were used. Sample preparation for flow cytometry was started by incubating a living individual for 2 min in a solution of 2% N-acetyl-L-cysteine at pH 7 to remove the mucus and thus prevent the formation of cell aggregates in the subsequent steps. Then, the animal was washed using a mixture of tap and distilled water (1:1) and subsequently placed in 1 ml of maceration solution composed of distilled water, glacial acetic acid and glycerol (13:1:1) and incubated for 15 min at room temperature. After the incubation, the cells were separated by gently pipetting using a cut tip and filtered through a nylon mesh with a pore size of 75 μm. Finally, the macerated cell suspension was stained for 5 min with 6 μl of Hoechst (stock 1 mg/ml), and the quantity of DNA was measured with a Gallios Flow Cytometer at the Unitat de Citometria dels Centres Científics i Tecnològics de la UB (CCiT, UB). To characterize the ploidy of an individual by flow cytometry, we first measured the cell suspension of that sample and counted the peaks observed (indicator of cell populations with different DNA content) and the fluorescence mode value of each peak. Then, we measured and annotated the same parameters for a cell suspension corresponding to a triploid control individual from the well-characterized population of Santa Fe del Montseny (Catalonia) [40], which we also karyotyped to verify its published ploidy. Finally, we analyzed a mix of the two suspensions thus allowing a direct comparison of both (Additional file 2: Figure S1). We used the latter values to infer the ploidy of the query individuals. This approach was conducted to avoid putative differences in the fluorescence values between samples due to slightly differences in the pH or in the duration of the staining.

### DNA sequence data

Individual total genomic DNA was extracted from the 32 ethanol-fixed specimens using the commercial reagent DNAzol (Molecular Research Center Inc., Cincinnati, OH) by following the manufacturer’s instructions. Two genomic regions were PCR-amplified for all the individuals: a fragment of the nuclear gene *Transmembrane p24 trafficking protein 9* (TMED9) and a fragment of the mitochondrial gene *Cytochrome oxidase subunit 1* (Cox1). The TMED9 gene was selected because it is a single copy gene in the species *Schmidtea mediterranea* containing a long intronic region (total amplified exonic region: 197 bp; total amplified intronic region: 751 bp) [43, 44]. The conditions for the PCR reactions for Cox1 were as previously published [45]. The amplification conditions for TMED9 were the following: 1) 2 min at 94 °C, 2) 45 s at 94 °C, 3) 50 s at 58 °C, 4) 40 s at 72 °C and 5) 3 min at 72 °C. Steps 2, 3 and 4 were run for 35 cycles. The primer sequences used to amplify each molecular marker are detailed in Additional file 1 Table S2.

All the PCR products were purified using a vacuum system (MultiScreen™ HTS Vacuum Manifold, Millipore Corporation, Billerica, MA, USA) and subsequently cloned using an HTP TOPO TA Cloning Kit for sequencing (Invitrogen, California, USA) following the manufacturers’ protocols. At least fifteen colonies per individual were amplified using the universal T3 and T7 primers. The sequencing reactions were run either in an automated sequencer (ABI Prism 3730) by the Unitat de Genòmica of Centres Científics i Tecnològics of the Universitat de Barcelona (CCiT, UB) or by Macrogen Corporation (Amsterdam, the Netherlands) using the same universal primers. Complementary strands of DNA were edited and assembled using Geneious version 10 [46].

### Sequence alignments and datasets

We aligned the sequences obtained for both genes at the nucleotide level using the online software MAFFT version 7 [47]. The alignments were cut at the same length using Geneious in order not to include missing data. The exonic and intronic regions of TMED9 were identified by comparing the gene with that of the annotated genome of *Schmidtea mediterranea* available online in SmedGD datababe [43, 48]. The reading frame of Cox1 and the coding regions of TMED9 were checked by translating the nucleotides into amino acids in Geneious. For Cox1 we used the GenBank genetic code Table 9 (mitochondrial echinoderm), while for TMED9 we used the genetic code Table 1 from GenBank (standard).

In a cloning experiment, DNA polymerases can introduce errors in the sequences during the first PCR (amplification of the target gene for each individual), during the cloning PCR (amplification of each clone), or in the sequencing reaction. Errors due to polymerase mistakes during the cloning PCR or due to sequencing errors can be generally detected as double peaks in the chromatograms. However, polymerase mistakes during the first PCR cannot be detected in the chromatograms. To evaluate the impact of polymerase errors in our data, we calculated the average number of mutations per haplotype that could be due to polymerase errors during the first PCR of the cloning process using the error rate of *Taq* DNA polymerase (2.28 × 10^− 5^) implemented in the PCR fidelity calculator web tool provided by Thermo Fisher [49]. For the nuclear marker TMED9, it was estimated that 75.65% of the PCR products would contain a single error due to polymerase mistakes, while for Cox1 only 59.18% of the PCR products would have a single error. To mitigate the effects of those artifact mutations, as recommended ([50], and references therein), we identified the singleton sequences of each individual that were separated by a single point mutation from other nonsingleton sequences and recoded them as the latter (Additional file 2 Figure S2). For the nuclear gene, we identified an average of 5 sequences per individual as being possible results of polymerase errors. Therefore, we recoded them. In the case of Cox1, only an average of 3 sequences per individual were identified as being a possible result of polymerase errors and were subsequently recoded.

### Intraindividual genetic diversity and effect of selection

We calculated the intraindividual number of different TMED9 alleles and Cox1 haplotypes using the program DnaSP v5 [51]. We also used DnaSP v5 to calculate the intraindividual genetic diversity at both the haplotype (*H*_D_) and nucleotide levels (π) for the two gene fragments and the intraindividual proportion of synonymous mutations (Ks), nonsynonymous mutations (Ka) and the ratio Ka/Ks (Ω). To test for significant differences in the estimates of genetic diversity and selection parameters between individuals depending on their reproductive strategy, we conducted an analysis of variance for each estimated parameter using the program Past3 [52]. We used one-way ANOVA Tests followed by Tukey’s pairwise comparison for the normal variables, the nonparametric Kruskal–Wallis test followed by Dunn’s post hoc pairwise comparison for the non-normal variables and Welch’s F test for variables with unequal variances. All *p*-values obtained in the pairwise comparisons were corrected for multiple testing.

An analysis of molecular variation (AMOVA) was performed to see how the genetic variation was partitioned within the different reproductive strategies. AMOVA was conducted with the software ARLEQUIN 3.5.2 [53] using pairwise differences with 10,000 permutations and leaving the rest of the parameters at their defaults. We quantified how much variation was explained within the different reproductive strategies: (1) between populations, (2) between individuals within the same population, and (3) within individuals.

### Phylogenetic reconstructions and haplotype networks

We took two different approaches to analyze the genetic data under an evolutionary framework: phylogenetic inferences and haplotype networks. Phylogenetic reconstructions were used to give directionality to the evolutionary processes, while haplotype networks were used to study the relationship between the alleles (this last approach is especially suitable when diversification has occurred in a short period of time and both ancestral and descendant haplotypes exist at the same time). We inferred the haplotype networks for the two genes for each individual and at the species level using the program Network version 4.6 [54]. We first imported separately the alignments of each gene into DnaSP v5 to convert them into Roehl files to be later processed in Network. The networks were constructed using the median-joining method [55], taking into account the minimum-length connections between the sequences (ε parameter equal to zero).

We inferred the phylogeny of each gene using Bayesian inference (BI) and maximum likelihood (ML) methods. Two *Dugesia* species phylogenetically close to *D. subtentaculata* [41] were used as the outgroup: *D. hepta* Pala, Cassu & Vacca, 1981 and *D. benazzii* Lepori, 1951. The degree of saturation of each alignment was assessed with the software Dambe [56] using a test of substitution saturation [57, 58], which resulted in no saturation in either of the two molecular markers, as the index of saturation in both cases was significantly lower than the index of critical saturation (TMED9: Iss = 0.192, Iss.c = 0.820, *p*-value< 0.01; Cox1: Iss = 0.129, Iss.c = 0.804, p-value< 0.01). The best substitution model for each analysis was determined using jModelTest2 [59]. The Bayesian analyses were conducted with the program MrBayes 3.2 [60] with two runs of 5,000,000 generations with four chains and sampling at intervals of 2000 generations each. Convergence of the Markov chain Monte Carlo (MCMC) chains for the two runs was confirmed after checking that the standard deviation of split- frequencies reached a value below 0.01. To infer the best tree and posterior probabilities, the default burn-in of 25% was used after checking that the two runs had reached the stationary phase. The maximum likelihood phylogenetic inference was conducted using the program RaxML 7.0.3 [61]. Two independent analyses were performed with different strategies to obtain the support for the nodes, one using the rapid bootstrap algorithm with 2000 replicates and another one using the standard bootstrap algorithm with 1000 replicates.

## Results

### Ploidy level of the populations

The results of previous works showed that sexual populations of Soller, Calobra and Bosque were diploid, while the fissiparous population of SantaFe was triploid [40, 42]. The ploidy level inference for the rest of populations using flow cytometry resulted in the detection of different ploidies. The three analyzed individuals of the sexual population of Alte were diploid, while the three analyzed individuals of the sexual population of Artavia were triploid (Additional file 1: Table S3). The four analyzed individuals of the facultative population of Trelles were triploids, while in the facultative population of Hortas we found one diploid and one triploid individual (Hortas3S and Hortas4S, respectively). Finally, the five analyzed individuals from the fissiparous population of Estella were tetraploids, while the two analyzed individuals from the fissiparous population of Truchas were mixoploids (combining approximately a 35% of triploid cells and a 65% of tetraploid cells) (see Additional file 1: Table S3).

### Intraindividual genetic diversity

An average of 14 and 12 intraindividual sequences were obtained for the nuclear and the mitochondrial marker, respectively (Table 1), representing a total of 810 sequences analyzed. The 453 sequences obtained for the nuclear marker were 948 bp in length, while the 357 sequences obtained for the mitochondrial gene were 649 bp in length. The analyses performed with the program DnaSP v5 revealed a total of 209 different alleles for the nuclear gene and 52 different haplotypes for the mitochondrial gene for all the individuals analyzed in the present study (GenBank accession numbers in Additional file 1: Table S4 and Table S5). The number of different intraindividual nuclear alleles varied from 2 to 8 in individuals from sexual populations, from 5 to 12 in individuals from facultative populations and from 6 to 16 in individuals from fissiparous populations (Table 1). For the mitochondrial gene, the number of different intraindividual haplotypes varied from 1 to 4 in individuals from sexual populations and from 1 to 7 in the rest of the individuals (Table 1).

**Table 1:** Intraindividual number of cloned sequences and different haplotypes obtained for the two molecular markers

	TMED9		Cox1	
	N	h	N	h
Sexual				
Calobra1S	15	2	14	2
Calobra2S	15	6	8	2
Calobra3S	15	7	15	1
Soller1S	14	8	10	1
Soller2S	13	6	21	2
Soller3S	15	8	15	4
Bosque1S	15	6	12	2
Bosque2S	15	6	12	2
Bosque3S	15	6	14	3
Alte1	13	4	11	2
Alte2S	14	6	9	2
Alte3S	12	6	13	2
Artavia1S	11	7	7	1
Artavia2S	13	5	13	1
Artavia3S	15	6	14	2
Σ = 15	$\overline{\mathrm{x}}$=14	$\overline{\mathrm{x}}$=6	$\overline{\mathrm{x}}$=13	$\overline{\mathrm{x}}$=2
Facultative				
Hortas1A	15	10	7	1
Hortas2A	15	9	6	3
Hortas3S	12	8	5	1
Hortas4S	15	5	7	4
Hortas5S	14	8	14	3
Trelles1A	14	9	12	4
Trelles2	14	11	13	7
Trelles3S	15	12	13	5
Σ = 8	$\overline{\mathrm{x}}$=14	$\overline{\mathrm{x}}$=9	$\overline{\mathrm{x}}$=10	$\overline{\mathrm{x}}$=4
Fissiparous				
SantaFe1A	28	16	22	3
SantaFe2A	13	7	6	1
SantaFe3A	8	6	12	3
Truchas1A	14	8	15	6
Truchas2	12	12	13	7
Truchas3	14	7	13	3
Estella1A	12	9	4	1
Estella2A	13	9	3	1
Estella3	15	8	4	1
Σ = 9	$\overline{\mathrm{x}}$=14	$\overline{\mathrm{x}}$=9	$\overline{\mathrm{x}}$=10	$\overline{\mathrm{x}}$=3

N: number of cloned sequences per individual

h: number of obtained haplotypes per individual

**Fig. 3:**
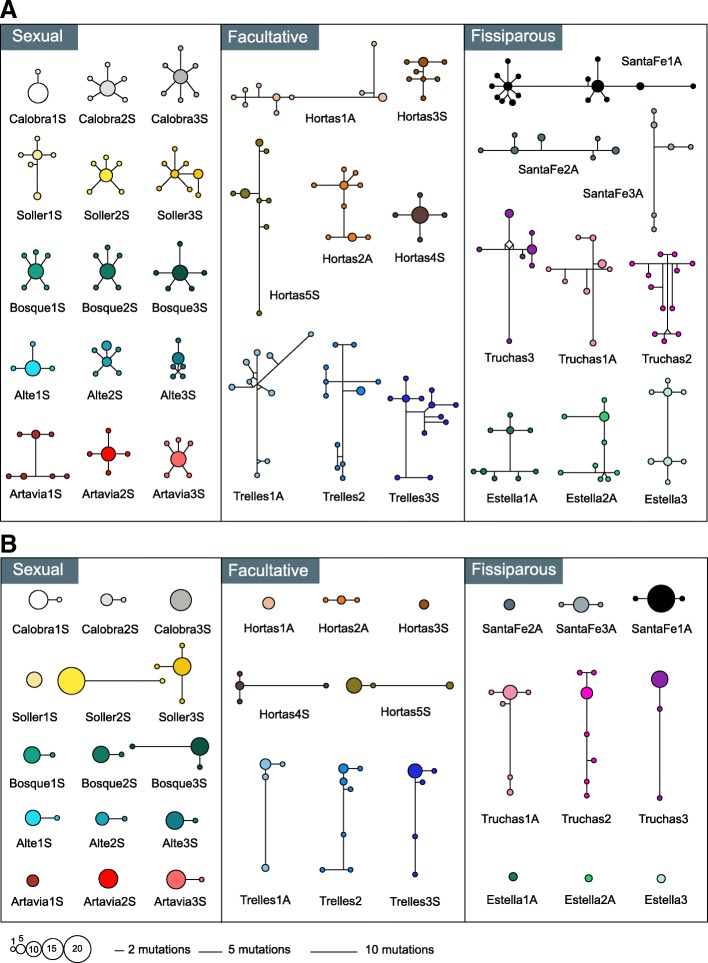
Intraindividual haplotype networks of the nuclear gene (**a**) and the mitochondrial gene (**b**). Each individual is depicted with a different color, and the code of the individual is given under its network. Each circle represents a different haplotype, and the size of the circle indicates the frequency of each haplotype within the individual. Branch lengths are proportional to the number of mutations

**Fig. 4:**
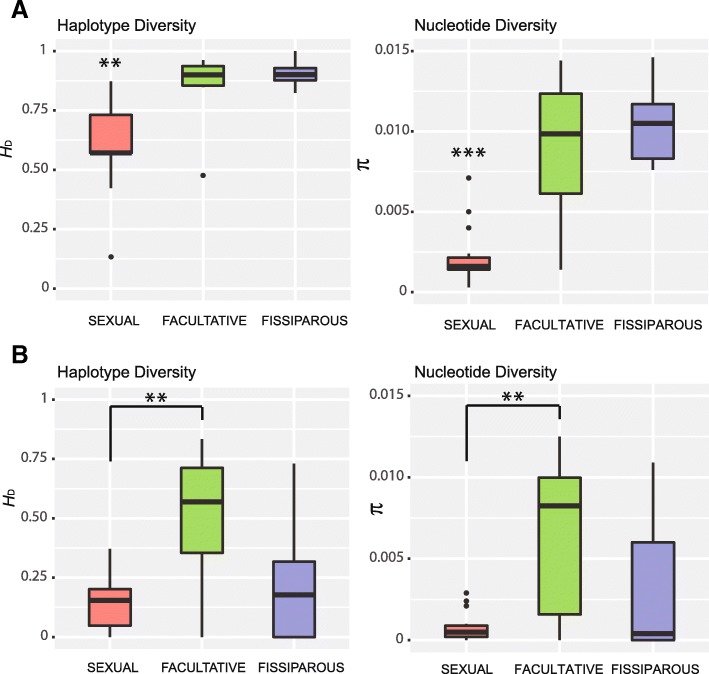
Graphical representation of the intraindividual haplotype and nucleotide diversity for each reproductive strategy, for the nuclear gene (**a**) and for the mitochondrial gene (**b**). Boxes are delimited by the first and third quartiles, and the median is represented by a thick line. Whiskers delimit the minimum and maximum nonoutlier values. Outlier values are represented by black dots. Asterisks indicate significant differences in the statistical comparison between reproductive strategies (*p = 0.01–0.05; ** *p* = 0.001–0.01; *** *p* < 0.001). See Additional file 1: Table S6 for genetic diversity values and Additional file 1: Table S7 for statistical comparisons

The intraindividual nuclear haplotype networks showed that 14 out of the 15 analyzed individuals from sexual populations presented a star-like pattern, consisting of one or two majoritarian alleles from which other, minority and closely related ones originated (Fig. 3a). On the other hand, 15 out of the 17 analyzed individuals from fissiparous and facultative populations showed a divergent pattern, consisting of many distantly related alleles occurring at similar frequencies (Fig. 3a). The star-like pattern of sexual individuals for the nuclear gene was characterized by statistically significantly lower *H*_D_ and π values compared to the divergent pattern of fissiparous and facultative individuals (Fig. 4a; see Additional file 1: Table S6 and Table S7).

At mitochondrial level, 12 of the analyzed sexual individuals exhibited a star-like pattern similar to that for the nuclear gene, except for three of them (Soller2S, Soller3S and Bosque3S) that showed a minority haplotype highly differentiated from the majority one (Fig. 3b). On the other hand, the six analyzed individuals from the fissiparous populations of Santa Fe and Estella plus three individuals of the facultative population of Hortas (Hortas1A, Hortas2A and Hortas3S) showed a star-like intraindividual pattern (like sexual individuals), while the three individuals from the fissiparous population of Truchas and the three individuals from the facultative population of Trelles showed a divergent pattern (Fig. 3b). The statistical comparisons of haplotype and nucleotide diversity between the different reproductive strategies at mitochondrial level showed that there were only significant differences in individuals from facultative populations compared to individuals from sexual populations (Fig. 4b; see Additional file 1: Table S6 and Table S7).

Finally, the intraindividual pattern of facultative populations either at nuclear or mitochondrial level was not correlated with the type of reproduction that the individuals showed at the moment of collection, since we found individuals reproducing sexually with a divergent pattern (e.g., Hortas5S) and fissiparous individuals with a star-like pattern (e.g., Hortas2A), and the other way around (Fig. 3).

### Analysis of molecular variation

**Fig. 5:**
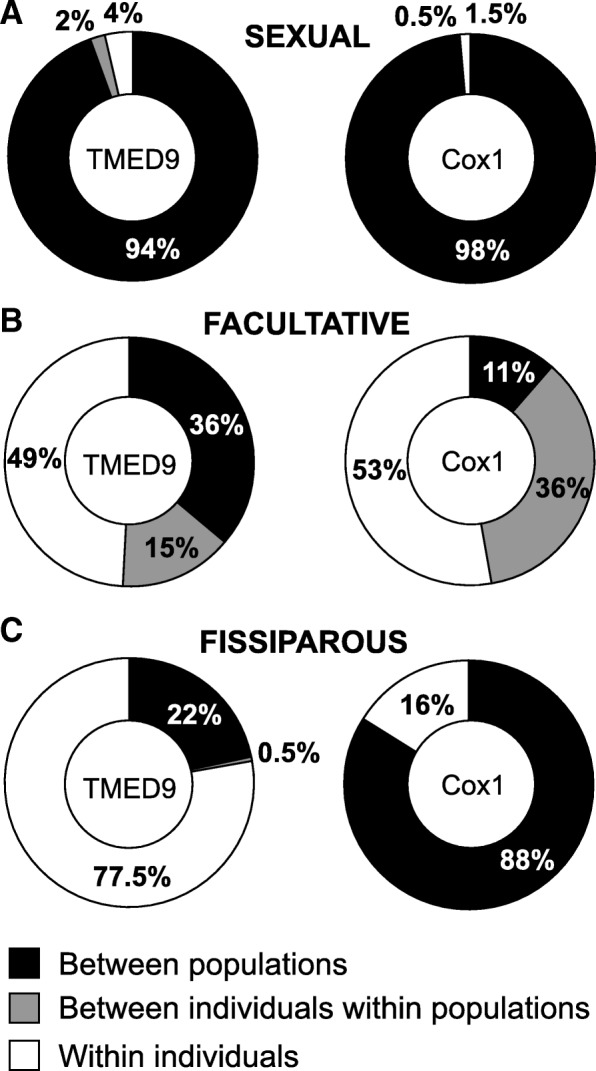
Graphical representation of the analysis of molecular variance (AMOVA) for each reproductive strategy and gene. Coloured squares indicate the percentage of genetic variation that is explained within each reproductive strategy; between populations, between individuals within populations and within individuals

The analysis of molecular variation (AMOVA) showed that the genetic variation in sexual populations was mostly explained by differences between populations (94% for the nuclear gene, 98% for the mitochondrial gene) (Fig. 5a). In facultative populations, half of the genetic variation was explained by differences within individuals (in both genes), while the rest of the genetic variation was explained by differences between populations and differences between individuals of the same population, in different proportions depending on the gene (Fig. 5b). Finally, the genetic variation in fissiparous populations was explained both by differences between populations and differences within individuals, in a different proportion depending on the gene. In the case of the nuclear gene a 77.5% of the genetic variation was explained by differences within individuals, while in the case of the mitochondrial gene a 88% of the genetic variation was explained by differences between populations (Fig. 5c).

### Effect of selection

**Fig. 6:**
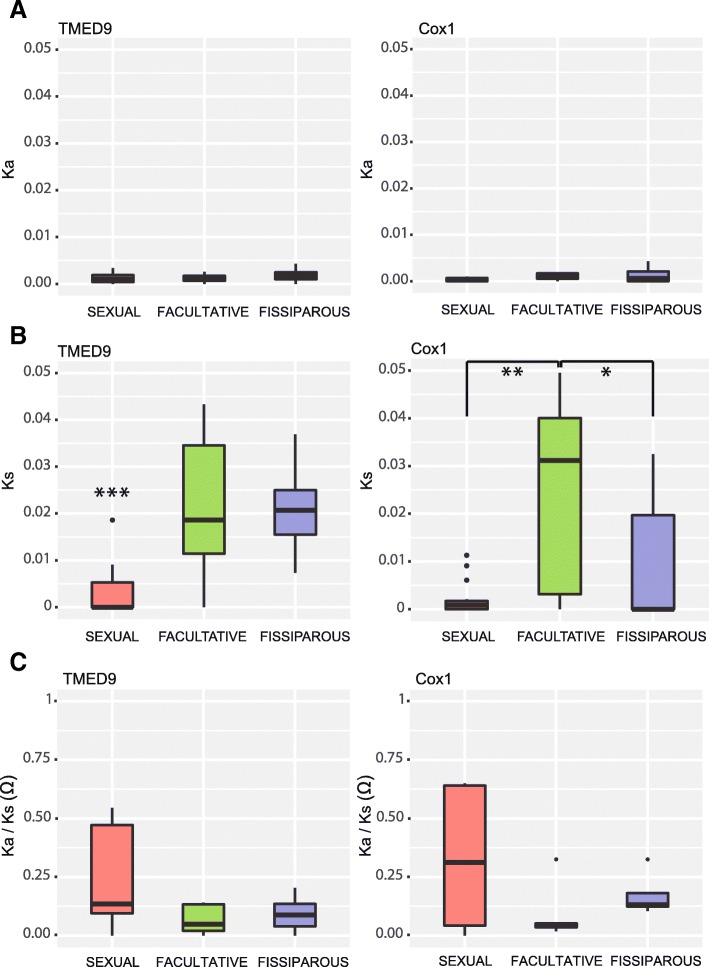
Graphical representation of the intraindividual proportion of nonsynonymous mutations (**a**), synonymous mutations (**b**), and the ratio Ka/Ks (**c**). Boxes are delimited by the first and third quartiles, and the median is represented by a thick line. Whiskers delimit the minimum and maximum nonoutlier values. Outlier values are represented by black dots. Asterisks indicate significant differences in the statistical comparison between reproductive strategies (*p = 0.01 0.05; ** *p* = 0.001–0.01; *** *p* < 0.001). See TableS6 for Ka and Ks values and Table S7 for statistical comparisons

The proportion of nonsynonymous mutations (Ka) for both genes within each individual was extremely low and not significantly different between the individuals of the three reproductive strategies when comparing the mean values obtained per reproductive strategy (Fig. 6a; see Additional file 1: Table S6 and Table S7). However, the mean intraindividual proportion of synonymous mutations (Ks) of the nuclear gene was significantly lower in individuals from sexual populations than in those from fissiparous and facultative populations (Fig. 6b; see Additional file 1: Table S6 and Table S7), an expected result given the low intraindividual π detected in this reproductive strategy. In the case of the mitochondrial gene, both individuals from sexual and fissiparous populations showed a significantly lower proportion of synonymous mutations (Ks) than individuals from facultative populations (Fig. 6b; see Additional file 1: Table S6 and Table S7). Finally, we found that there were no significant differences in the mean Ω value between the different reproductive strategies in any of the two gene fragments, which in all cases was lower than 1 (Fig. 6c; see Additional file 1: Table S6 and Table S7).

### Phylogenetic inferences and species haplotype networks

The phylogenetic analyses performed with Bayesian Inference and Maximum Likelihood (for both bootstrapping algorithms) yielded the same topology for each molecular marker (the supports for ML only varied to slightly higher supports when the rapid bootstrap strategy with more replicates was used). The sexual populations of Bosque, Calobra and Soller were recovered as the first to differ, forming three monophyletic clades (named as Sexual clades I, II and III, respectively) that were highly differentiated between themselves and from the rest of populations (see Additional file 2: Figure S3 and Figure S4). Their order of appearance remained elusive, as it was different in both genes and not fully supported in any of them. On the other hand, all fissiparous and facultative populations together with the sexual populations of Alte and Artavia conformed a derived and highly supported monophyletic group (referred from now on as Mixed clade), irrespective of the gene analyzed. Moreover, the nuclear genetic diversity of the Mixed clade was distributed into four main clades (Clades A, B, C and D), while its mitochondrial genetic diversity was distributed into six main clades (Clades 1, 2, 3, 4, 5 and 6) (see Additional file 2: Figure S3 and Figure S4).

**Fig. 7:**
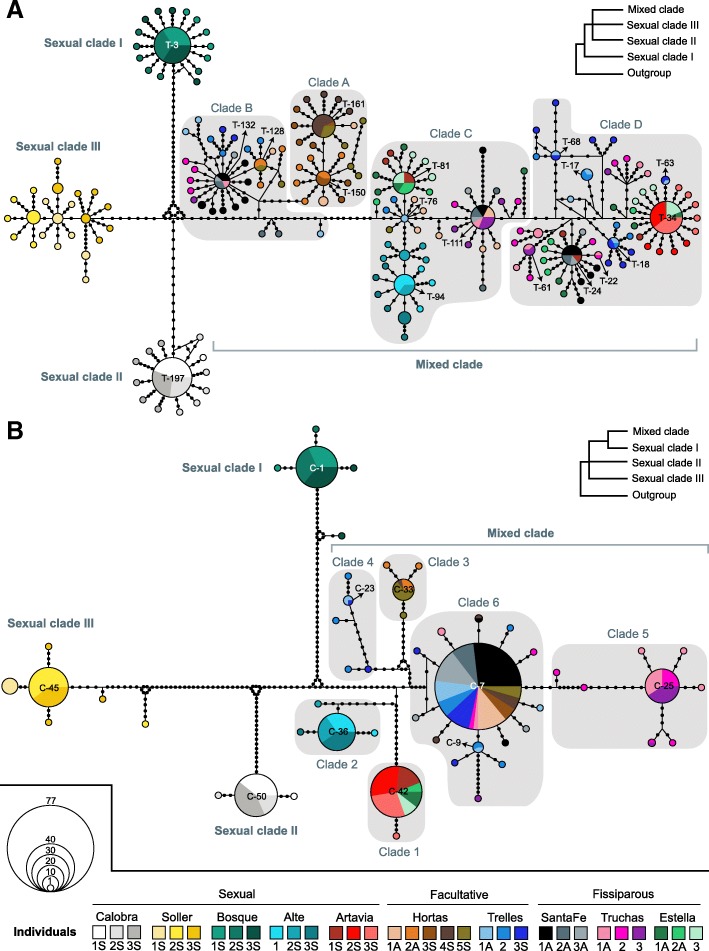
Species haplotype network for the nuclear gene (**a**) and for the mitochondrial gene (**b**). Each individual is depicted with a different color. Each circle represents a different haplotype, and the size of the circle is proportional to the frequency of each haplotype in the species. Mutations are depicted as small black dots. All shared haplotypes are named with their code (see Tables S4 and S5). For each gene, a schematic representation of the phylogeny is shown in the right top corner (see Additional file 2: Figures S3 and S4), nodes with low support values are depicted as polytomies

The species haplotype networks recovered the same main clades than the phylogenetic reconstructions (Fig. 7). They showed that while for the Sexual clades I, II and III ancestral alleles and haplotypes were lost, in the Mixed clade both derived and ancestral were present. For instance, in TMED9, alleles of clade D derived from certain alleles of C, and those of C derived from certain alleles of clade B (Fig. 7a). In the case of Cox1, haplotypes from clade 5 derived from the majoritarian haplotype of clade 6 (C-7, Fig. 7b), while clades 1, 2, 3 and 4 derived from a common ancestor with clade 6 (Fig. 7b). The two sexual populations within the Mixed clade (Alte and Artavia) only showed derived alleles and haplotypes. We found that the genetic diversity of all fissiparous and facultative individuals was distributed in both ancestral and derived clades at least in one of the two molecular markers (Fig. 7). Moreover, the derived mitochondrial genetic diversity was private of each population (the only haplotype shared between populations was the haplotype C-7 of clade 6), while both ancestral and derived nuclear genetic diversity was shared between individuals of different populations (Fig. 7). However, in the case of the nuclear marker, a maximum number of three alleles was shared between individuals of different fissiparous and facultative populations, while the number of shared alleles between the individuals of the same populations could be higher.

Importantly, the fact that both sexual and fissiparous individuals of each facultative population share the same ancestral and derived genetic diversity shows that they belong to a single genetic lineage, as expected for a population constituted by facultative individuals. This fact, together with our previous finding that some sexual individuals in these populations show a divergent haplotype pattern, demonstrate that these populations are actually facultative populations.

## Discussion

### High tissue turn-over and growth-degrowth dynamics as putative drivers of mosaicism in sexual planarians

Against our theoretical expectations, we found that all individuals from the sexual populations analyzed in the present study (excepting the individual Calobra1S) showed a higher number of nuclear alleles than those expected for their ploidy level, indicating that they are mosaics. Differing from most individuals from fissiparous and facultative populations, the genetic pattern of exclusively sexual individuals was characterized at both nuclear and mitochondrial level by one majority allele (generally shared with the other members of the population) and few derived low frequency alleles (private of each individual). This pattern indicates that the majoritarian allele of each individual was most probably sexually inherited, while the rest of low frequency alleles may be the result of somatic mutations.

It could be possible that some of the low-frequency alleles resulted from polymerase errors during the PCR reaction. Based on the error rate of the *Taq* polymerase (2.28 × 10^− 5^), we estimated that a 75% of the alleles could bear one artifactual mutation. Thus, we applied a widely accepted correction method to our dataset to minimize this effect. Nevertheless, we found that most of the cloned alleles of the same PCR product were differentiated by many more mutations than those expected by mistakes of the *Taq* polymerase. This suggests that although certain mutations are probably artificial, most of these low-frequency alleles would have originated due to other factors, such as somatic mutations.

The appearence of somatic mutations during the lifespan of individuals has been detected in several sexual organisms, including humans [62, 63], and it can be due to a wide range of causes, such as DNA damage due to environmental factors or replication errors during tissue homeostasis, among others ([64], and references therein). Planarians, moreover, have an extraordinary cell turn-over rate during their normal tissue homeostasis. Neoblast division and differentiation of the neoblast’s progeny have been found to occur on an ongoing basis in nongrowing individuals ([65], and references therein). Additionally, conditions of severe starvation induced by the lack of food result in massive degrowth of the planarian body due to an increase in the cell death of differentiated cells. This situation is easily reversed by the recovery of food sources, which results in a neoblast-driven growth of the individual [25, 66]. Therefore, a potentially high tissue turnover, in addition to the growth-degrowth cycles that take place in natural conditions, may be responsible for the intraindividual genetic pattern observed in most of the sexual individuals.

### Intraindividual genetic diversity of fissiparous planarians: evidence for a *mosaic Meselson effect*

In the case of fissiparous populations, we initially predicted the existence of mosaicism and the Meselson effect within individuals, due to the progressive accumulation of somatic mutations in the neoblasts over generations of fissiparous reproduction. The intraindividual nuclear divergent pattern (characterized by significantly higher levels of haplotype and nucleotide diversity compared to sexuals; Figs. 3 and 4) together with the high number of alleles (Table 1) observed in most individuals from fissiparous populations gives support to that hypothesis. Surprisingly, we did not expect to find equal levels of intraindividual genetic diversity in individuals from fissiparous and facultative populations, since we expected that the bottlenecks that represent the pass through a one celled zygote state plus recombination during sexual events, would reduce both the degree of mosaicism and the genetic differentiation between alleles in the facultative populations. These findings suggest that fissiparous reproduction may be the predominant type of reproduction in the facultative populations studied. Notably, the levels of intraindividual haplotype and nucleotide diversity reported in most fissiparous and facultative individuals analyzed in the present study are so extremely high that they are comparable, at mitochondrial level, to the highest levels found between different individuals of parthenogenetic populations of different taxa (Table 4 in [8]).

A potential caveat to our hypothesis of mosaicism and the Meselson effect within fissiparous and facultative individuals is the possible existence of paralog nuclear genes and numts (i.e., mitochondrial copies in the nucleus). If part of this genetic diversity corresponded to paralogs or numts, we would expect that they would be equally present in individuals from sexual populations. Particularly, in individuals from sexual populations derived from fissiparous ones, such as Alte and Artavia. Nevertheless, they are not only absent from sexual populations in general but also from individuals from Alte and Artavia. A second caveat, as previously mentioned for sexual populations, is that some mutations of the alleles and haplotypes of fissiparous and facultative individuals could be due to mistakes in the activity of the polymerase. Nevertheless, they could only explain a minority of the intraindividual genetic diversity detected in these reproductive strategies, since most of these alleles and haplotypes are shared between different individuals or are so highly differentiated that it is extremely improbable that all mutations are a consequence of polymerase mistakes (Fig. 7). Therefore, the existence of mosaicism combined with the Meselson effect remains as the most plausible explanation to interpret the intraindividual genetic pattern found in most of the fissiparous and facultative individuals analyzed in the present study.

Importantly, given that the number of highly differentiated nuclear alleles is, for most individuals, higher than its ploidy, we can deduce that these highly differentiated alleles are distributed across different cells, in contrast to what is found when the Meselson effect occurs in parthenogenetic individuals. We propose this variation of the Meselson effect to be referred as the *mosaic Meselson effect*, which we define as the existence of a genetically heterogeneous cell population within the body of an organism, carrying highly divergent alleles in homologous genetic regions. Evidence for the occurrence of this effect in other fissiparous *Dugesia* species can be found in two studies that were focused on *D. sicula* and *D. japonica* [67, 68]*.* Moreover, although this is the first time that the mosaic Meselson effect is suggested, it may also occur in other fissiparous metazoans such as star-fish and corals [18], and in long-lived plants where genetic differences between branches within individuals have been studied but not quantified from a Meselson effect point of view [69–71]. At the mitochondrial level, whether the highly divergent haplotypes of the individuals are mainly found in different cells (mosaicism) or in the same cell will be discussed below.

**Fig. 8:**
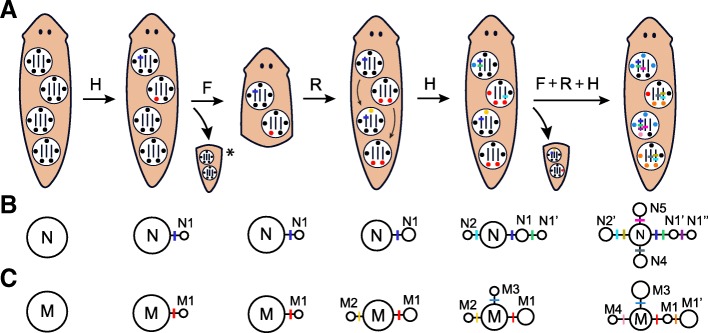
Schematic representation of the mosaic Meselson effect occurring in a planarian during fissiparous reproduction. **a** Starting from a homozygous triploid individual having four neoblasts (each with three alleles and five mitochondria), mutations (depicted by different colors) start to appear randomly in the alleles and mitochondria of the neoblasts. After several cycles of homeostasis (H), fission (F) and regeneration (R), the fissiparous individual shows an increased number of derived alleles and haplotypes in different cells but still shows the ancestral. **b** and **c** depict the nuclear and mitochondrial haplotype networks at each state, N and M are the ancestral allele and haplotype, the derived alleles and haplotypes are indicated by subsequent numbering and indexing of N and M. * The same processes would happen in both pieces after a fission event

Interestingly, the evolutionary analysis revealed that the intraindividual genetic diversity of fissiparous and facultative individuals was characterized by the existence of a mix of ancestral and derived alleles at both the nuclear and mitochondrial levels (Fig. 7). The capacity of retaining or “freezing” genetic diversity has already been proposed to occur in parthenogenetic lineages, conferring them a clear advantage compared to sexual lineages when genotypes are well adapted to the environmental conditions [72, 73], and also explaining why the genetic differentiation between parthenogenetic populations is generally lower than between sexual populations [6]. However, differing from parthenogenetic organisms, fissiparous reproduction may allow individuals to keep accumulating somatic mutations in some cells under this general “frozen” state, explaining why they can show a mixture of ancestral and derived genetic diversity. Therefore, the mosaic Meselson effect due to fissiparous reproduction may not only be characterized by the existence of highly divergent alleles in a mosaic context but also for the coexistence of ancestral and derived genetic diversity within individuals. In Fig. 8 it is depicted how the synergic effect of fissiparous reproduction and tissue homeostasis may result in this special pattern of intraindividual genetic diversity. Further investigations, based on a genomic approach, and including regional analyses of different body regions and organs, or even studying them at the single cell level, would be of great value to improve our understanding on the intraindividual genetic characteristics of these organisms.

### Intraindividual selection in fissiparous planarians

Considering the evidence for the existence of mosaicism combined with the Meselson effect in fissiparous and facultative populations, we also expected to find a high incidence of Muller’s ratchet in individuals from these populations. But, contrary to our expectations, our results suggest that purifying selection is occurring in all individuals analyzed in the present study (Fig. 6).

These results point out that intraindividual cell selection could be responsible for the elimination of deleterious mutations during periods of fissiparous reproduction. Processes of intraindividual cell selection are well known to occur in insects and mammals due to different mechanisms ([74], and references therein) and their consequences have been analyzed theoretically [75, 76] and empirically observed in some plants, i.e., eucalyptus trees that present one or a few branches resistant to a plague as a result of the selection of a somatic mutation [70, 77]. In our case, the Cox1 protein is known to be essential for the viability of any cell. Therefore, deleterious mutations in Cox1 would result in the elimination of the mutated mitochondria or even the whole cell bearing it, resulting in intraindividual selection. However, in the case of the nuclear gene TMED9, it is known to code for a transmembrane protein but its exact function in planarians is not yet characterized. Hence, although our results seem to indicate that it is also under purifying selection and in consequence it may also play an important role in neoblast survival, more data on nuclear genes is needed to confirm the existence of purifying selection in the nucleus at cellular level during fissiparous periods.

A test to our hypothesis on the existence of intraindividual purifying selection in mosaic fissiparous planarians could be to analyze genes that are only expressed in certain cell types or organs in individuals who have long been reproducing by fission (such as in individuals from the population of Truchas). In this case, we would expect that all cells that do not need the expression of those genes (including neoblasts) will be able to carry mutated nonfunctional or less efficient copies, and we would detect a Muller’s ratchet effect. Indirect evidence for the expected result of this test comes from a comparison of genomic and transcriptomic data from a laboratory lineage of *D. japonica* derived from a single individual that kept undergoing autonomous fission for over 20 years [68], where they detected that a 74% of the genes presented nonsynonymous polymorphisms. Nonetheless, another possibility exists to explain the lack of Muller’s ratchet in the two genes analyzed in the present study: the synergic action of intraindividual selection and occasional sex (see below).

### Multiple transitions between sex and fissiparity in the evolutionary history of *D. subtentaculata*

The topology of the phylogenetic trees, showing that the first lineages to diverge in *D. subtentaculata* are three sexual diploid populations while most populations within the monophyletic Mixed clade are triploid fissiparous or facultative (see Additional file 2: Figure S3 and Figure S4), suggests that the origin of the Mixed clade was due to a triploidization event from sexual diploid ancestors. This triploidization event possibly promoted a shift from sexual to fissiparous reproduction in the lineage that gave rise to the Mixed clade, since in the genus *Dugesia* polyploidy is highly associated with fissiparity [41]. Moreover, the evolutionary analyses also revealed that all individuals of the Mixed clade showed their nuclear genetic diversity distributed in the same ancestral and derived clades, indicating that after the above mentioned triploidization event, the ancestors of the Mixed clade reproduced by fission for a long time. However, the fact that most individuals of different populations nowadays share different combinations of at maximum three alleles, either ancestral or derived, can only be explained if different sexual events occurred after this period of fissiparous reproduction.

**Fig. 9:**
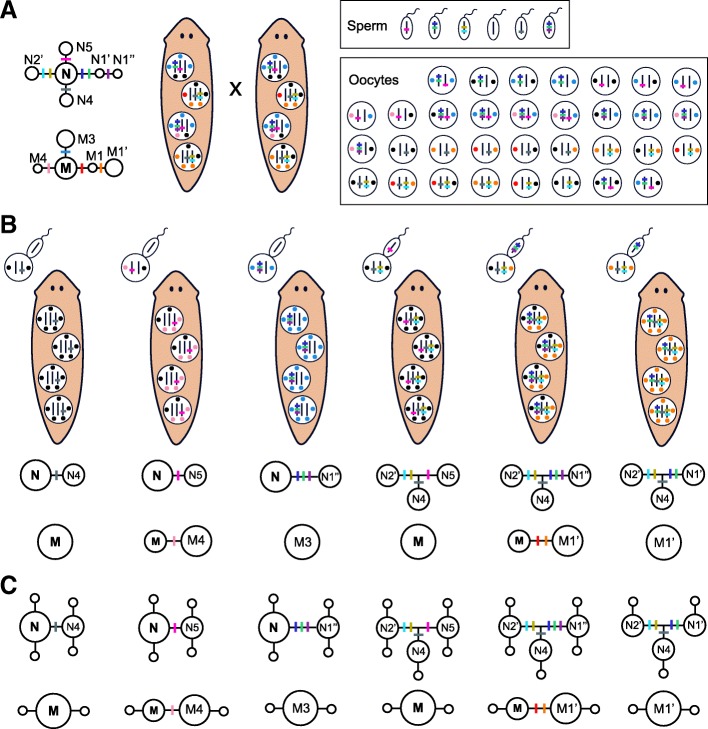
Schematic representation of new genetic combinations resulting from the cross of two resexualized fissiparous planarians. **a** When fissiparous individuals showing the mosaic Meselson effect resexualize, their original (depicted in black) and new alleles and haplotypes (depicted with different colors) segregate in their gametes. While haploid sperm only carries a single nuclear allele, diploid oocytes carry two alleles and two mitochondria (the bottleneck of mitochondria during oogenesis is represented by the loss of three mitochondria per neoblast). **b** After crossing, non-mosaic descendants are born showing different combinations of ancestral (N and M) and derived (indicated by a N or M with subsequent numbering and indexing) alleles and haplotypes, such as the six different genetic combinations shown in the figure as an example. Notice that derived alleles that progenitors had in different cells (such as N1’’ and N2’) can be inherited together in the offspring. **c** If descendants resume fissiparous reproduction, they will result in new lineages that accumulate new mutations in the alleles and haplotypes that they inherited

In resexualized fissiparous planarians, with the germ line appearing de novo from a population of genetically diverse neoblasts (due to the mosaic Meselson effect, Fig. 8), processes of segregation (Fig. 9a) and outcrossing (Fig. 9b) may result in descendants showing different combinations of ancestral and derived alleles but with a maximum number of shared nuclear alleles equal to their ploidy level. At the mitochondrial level, descendants could only inherit the haplotypes present in the neoblast precursor of the oocyte [78, 79], indicating that the progenitors of the individuals showing both ancestral and highly derived mitochondrial haplotypes were heteroplasmic at intracellular level. Nevertheless, the finding that individuals of different fissiparous and facultative populations don’t share any derived mitochondrial haplotype but they share the ancestral haplotype of the group (C-7 in Fig. 7b), indicates that their ancestors were also mosaics at the mitochondrial level, as we predicted with the mosaic Meselson effect. Therefore, processes of segregation and outcrossing (the last only for the nuclear genome) during sexual events in the fissiparous ancestors of the Mixed clade allow us to explain why some fissiparous and facultative individuals show a nuclear divergent pattern but a mitochondrial star-like pattern (e.g., individuals from Santa Fe in Fig. 3), or the other way around (e.g., individual Hortas4S in Fig. 3) (Fig. 9), explaining also how the haplotype and nucleotide diversity of fissiparous individuals could be significantly different from sexual individuals only at nuclear level. If the offspring after such sexual events resume fissiparity (Fig. 9c), new mutations can start to accumulate from the different combinations of inherited alleles and haplotypes, resulting in the patterns of shared and private intraindividual genetic diversity that we have observed in the different lineages (Fig. 7).

Although our results indicate that all populations of the Mixed clade share a common evolutionary history of fissiparous reproduction with facultative sex, we found that there have been posterior transitions between reproductive strategies. On the one hand, two populations returned to strict sex: the diploid population of Alte and the triploid population of Artavia. In both cases the return to strict sex promoted the recovery of the star-like intraindividual genetic footprint characteristic of sexual individuals. The rise of diploid sexual offspring from the outcrossing of two triploid resexualized fissiparous individuals has been directly documented under laboratory conditions in the species *D. ryukyuensis* [80]. Differing from Alte, the return to strict sexuality in the population of Artavia occurred without a change in the ploidy level. Interestingly, the existence of one individual of Artavia (Artavia1S) showing a slightly divergent pattern at the nuclear level (Fig. 3), suggests a recent return to sexuality from its fissiparous past. On the other hand, the three fissiparous populations analyzed in the present study seem to have become strict fissiparous. Individuals of these fissiparous populations have already started to generate new genetic diversity from the alleles and haplotypes that sexually inherited, but they are still genetically very similar to the other individuals of its own population (Fig. 5c). Finally, although our results indicate that fissiparous reproduction is the predominant reproductive strategy in the facultative populations we have studied, we found that present events of sex in these populations may enable the existence of an additional component of genetic diversity: differentiation between individuals (Fig. 5b), which is not found in populations where sex and fission is not regularly alternated. Different proportions of fissiparity and sex could then be selected under different ecological-climatic conditions, providing this species with a huge range of strategies to face them, as has been proposed in facultative populations of the sexual-parthenogenetic *S. polychroa* [81].

### Evolutionary advantages of fissiparous reproduction with occasional sex

The evolutionary model based on the combination of fissiparous reproduction with different rates of occasional sex that we have proposed represents a paradigmatic mode of evolution for several reasons. While the somatic genetic diversity may be partially hidden from selection when fission is the only way to reproduce, the recovery of sexuality may expose this genetic diversity to selection in the offspring in different combinations (depending on the segregation and out-crossing, Fig. 9b). Therefore, besides the possible processes of intraindividual selection at the neoblast level during fissiparous reproduction (that might eliminate extremely deleterious variants), events of occasional sex not only may help to prevent the occurrence of Muller’s ratchet in predominantly fissiparous linages ([82], and references therein) but also may promote a rapid adaptation to different environmental conditions. Moreover, the presence of this high intraindividual genetic diversity due to fissiparous reproduction may allow them also to overcome evolutionary problems such as those generated by population bottlenecks (a situation that, for instance, species of *Dugesia* endure each year in the Mediterranean region), since they will not result in a loss of genetic diversity. All these reasons could help explain the evolutionary success of the genus *Dugesia*, with more than 85 species distributed in Africa, Asia and Europe [83], compared to its sister genera *Schmidtea* [45] and *Recurva* [84], which principally reproduce sexually or by parthenogenesis and at present include only 4 and 2 species, respectively, distributed only in Northern Africa and Europe.

## Conclusions

The intraindividual genetic data obtained in the present study provide evidence for the existence of mosaicism combined with the Meselson effect (the mosaic Meselson effect) in fissiparous metazoans, specifically in planarians, an organism of complex tissue architecture. Furthermore, our results point out that the mosaic Meselson effect enables the existence of both ancestral and highly derived genetic diversity within the same individual, a genetic characteristic never described before and very interesting under an evolutionary point of view. Concomitantly, our results provide evidence that this special genetic diversity acquired during periods of fissiparous reproduction can be transmitted to the offspring through sexual events, allowing the generation of progeny with a huge range of genetic diversity and providing a scenario of possible multilevel selection (at both intraindividual and individual level). Further investigations using *D. subtentaculata* as a model organism, which may go to the genomic level, would be of great value to understand how fissiparous organisms can orchestrate such a genetically heterogenic cell population, including putative processes of intraindividual selection. Moreover, due to the impressive plasticity of this species in shifting ploidy and reproductive strategies, it can also be a good model to analyze the mechanisms that trigger polyploidization and how they are linked to the reproductive mode and environmental conditions. Nature is full of exceptions to our laboratory model organisms, and we need to study them to understand how they evolve and succeed.

## Additional files

Additional file 1: **Table S1.** List of populations used in this study, including information on the reproductive strategy, locality and collectors. **Table S2.** Sequence, source and annealing temperature of the primers used in this study. **Table S3.** Results of the ploidy inference by flow cytometry. **Table S4.** List of the different TMED9 alleles obtained in this study. The individuals showing each haplotype and its corresponding GenBank accession number are indicated. **Table S5.** List of Cox1 haplotypes, with the individuals showing each haplotype and its corresponding GenBank accession number. **Table S6.** Genetic diversity and proportion of synonymous and nonsynonymous sites at intraindividual level for the two molecular markers analyzed in the present study. **Table S7.** Results of the statistical tests used to compare the intraindividual mean levels of genetic diversity and types of mutations between the different reproductive strategies. (PDF 407 kb)

Additional file 2: **Figure S1.** Workflow of the ploidy level estimation using flow cytometry. 1) Cutting the animal in two pieces, 2) cell maceration, 3) DNA staining, 4) fluorescence measurement with a Gallios flow cytometer and 5) unknown-ploidy estimation by comparison with control. **Figure S2**. Approach used in this study for the correction of the putative artifactual mutations due to polymerase mistakes. In parentheses is indicated the frequency of each haplotype within the individual before and after the correction. **Figure S3.** Bayesian inference tree of all the alleles of the nuclear gene TMED9. The colors of the terminal branches indicate to which individual each allele belongs. The color of the outer circle indicates the reproductive strategy of each individual. Numbers at the nodes indicate the support values for the Bayesian inference (posterior probability)/the maximum likelihood (bootstrap). Bootstrap values correspond to the maximum likelihood analysis conducted with rapid bootstrap. Support values lower than 0.8 (posterior probability) and 75% (bootstrap) are represented with a -. Scale bar indicates the number of substitutions per site. **Figure S4.** Bayesian inference tree of all the haplotypes of the mitochondrial gene Cox1. Haplotypes of the same individual are pictured with the same color. Numbers in white indicate the different clades within the Mixed clade. Numbers at the nodes indicate the support values for the Bayesian inference (posterior probability)/the maximum likelihood (bootstrap). Bootstrap values correspond to the maximum likelihood analysis conducted with rapid bootstrap. Support values lower than 0.8 (posterior probability) and 75% (bootstrap) are represented with a -. Scale bar indicates the number of substitutions per site. (PDF 878 kb)

## Abbreviations

AMOVA: Analysis of molecular variation
BI: Bayesian inference
CCiT: Unitat de Citometria dels Centres Científics i Tecnològics
Cox1: Cytochrome oxidase subunit 1
MCMC: Markov chain Monte Carlo
ML: Maximum likelihood
TMED9: Transmembrane p24 trafficking protein 9
UB: Universitat de Barcelona
