# Supplementary material for: Outstanding intraindividual genetic diversity in fissiparous planarians (Dugesia, Platyhelminthes) with facultative sex
Source: BMC Evol Biol. 2019 Jun 20;19:130. doi: 10.1186/s12862-019-1440-1 (PMC6587288; doi:10.1186/s12862-019-1440-1)
Supplement: Supplementary file 1 — Table S1. List of populations used in this study, including information on the reproductive strategy, locality and collectors. Table S2. Sequence, source and annealing temperature of the primers used in this study. Table S3. Results of the ploidy inference by flow cytometry. Table S4. List of the different TMED9 alleles obtained in this study. The individuals showing each haplotype and its corresponding GenBank accession number are indicated. Table S5. List of Cox1 haplotypes, with the individuals showing each haplotype and its corresponding GenBank accession number. Table S6. Genetic diversity and proportion of synonymous and nonsynonymous sites at intraindividual level for the two molecular markers analyzed in the present study. Table S7. Results of the statistical tests used to compare the intraindividual mean levels of genetic diversity and types of mutations between the different reproductive strategies. (PDF 407 kb) [file 12862_2019_1440_MOESM1_ESM.pdf]

Table S1. List of populations used in this study, including information on the reproductive strategy, locality and collectors.

Population code	Reproductive strategy	Locality	Coordinates	Collectors
Calobra	Sexual	Sa Calobra, Mallorca, Balearic Islands, Spain	39.82932N 2.81538E	M. Vila-Farré
Soller	Sexual	Soller, Mallorca, Balearic Islands, Spain	39.75693N 2.71193E	M. Vila-Farré
Bosque	Sexual	El Bosque, Andalucía, Spain	36.76123N 5.50581W	M. Àlvarez, M. Riutort and L. Leria
Alte	Sexual	Alte, Algarve, Portugal	37.23827N 8.17199W	A.Valls, E.Solà, M.Riutort and L.Leria
Artavia	Sexual	Artavia, Navarra, Spain	42.74488N 2.08957W	M. Vila-Farré, E.Solà and L.Leria
SantaFe	Fissiparous	Santa Fe del Montseny, Catalonia, Spain	41.77356N 2.46638E	M. Vila-Farré, E.Solà and L.Leria
Truchas	Fissiparous	Peralejos de las Truchas, Castilla La Mancha, Spain	40.59318N 1.92561W	M. Vila-Farré, E.Lázaro and L.Leria
Estella	Fissiparous	Estella, Navarra, Spain	42.6746N 2.03519W	M. Vila-Farré, E.Solà and L.Leria
Hortas	Facultative	Portalegre, Portugal	39.3232N 7.40911W	M. Àlvarez, M. Riutort and L. Leria
Trelles	Facultative	Trelles, Asturias, Spain	43.476N 6.73358W	M. Vila-Farré, E.Solà and L.Leria

Table S2. Sequence, source and annealing temperature of the primers used in this study.

Gene	Primer	Sequence 5'-3'	Source	Annealing Temp (ºC)
Cox1	BarT (F)	ATGACDGCSCATGGTTTAATAATGAT	Álvarez-Presas *et al*. 2011	43
	COIR (R)	CCWGTYARMCCHCCWAYAGTAAA	Lázaro *et al.* 2009	45
TMED9	Dunuc12_1F (F)	CTCGTATCTCTGAATCTAGCCTC	Leria *et al.* 2019	53
	Dunuc12_1R (R)	GTTCATACAACTCAT TCT TC	Leria *et al.* 2019	46

Table S3. Results of the ploidy inference by flow cytometry.

Population	Sample	Number of peaks	Number of cells	Fluorescense	Proportion	Ploidy
**Alte (sexual)**	Control 1	1	5706	664		
	Alte1 [a]	1	5241	414		
	Control 1 + Alte1 [a]	2	12972 and 5450	378 and 593	1'91	**2n**
	Control 1	1	11120	656		
	Alte1S [a]	1	3425	429		
	Control 1 + Alte1S [a]	2	10170 and 12652	425 and 652	1'95	**2n**
	Alte2S [a]	1	11222	421		
	Control 1 + Alte2S [a]	2	3161 and 2134	451 and 678	1'99	**2n**
**Artavia (sexual)**	Control 2	1	15865	684		
	Artavia1S [a]	1	7363	687		
	Control 2 + Artavia1S [a]	1	24092	773	3	**3n**
	Artavia2S [a]	1	10876	685		
	Control 2 + Artavia2S [a]	1	6353	697	3	**3n**
	Control 3	1	5027	665		
	Artavia3S [a]	1	8419	673		
	Control 3 + Artavia3S [a]	1	11821	746	3	**3n**
**Hortas (facultative)**	Control 4	1	11160	595		

	Hortas3S [a]	2[b]	1443 and 5388	277 and 509		
	Control 4 + Hortas3S [a]	3[b]	6151, 15910 and 6042	242, 439 and 620	1'17 and 2'12	**2n**
	Hortas4S [a]	1	5234	562		
	Control 4 + Hortas4S [a]	1	18673	638	3	**3n**
**Trelles (facultative)**	Control 5	1	12612	602		
	Trelles_1	1	8688	562		
	Control 5 + Trelles_1	1	20985	602	3	**3n**
	Trelles_2	1	20521	570		
	Control 5 + Trelles_2	1	20594	602	3	**3n**
	Control 6	1	10771	574		
	Trelles_3	1	17734	576		
	Control 6 + Trelles3	1	23866	612	3	**3n**
	Trelles_4	1	16609	579		
	Control 6 + Trelles_4	1	23399	603	3	**3n**
**Truchas (fissiparous)[c]**	Control 7	1	4790	204		
	Truchas_1	2	1371 and 2451	633 and 805		
	Control 7 + Truchas_1	2	4495 and 1700	692 and 879	3 and 3'8	**3n and 4n**
	Control 8	1	5716	617		
	Truchas_2	2	1431 and 2682	718 and 895		

	Control 8 + Truchas_2	2	4403 and 2643	651 and 880	3 and 4'05	**3n and 4n**
**Estella (fissiparous)**[c]	Control 9	1	5251	178		
	Estella_1	1	1805	266		
	Control 9 + Estella_1	2	1109	304 and 437	4'31	**4n**
	Control 10	1	4706	347		
	Estella_2	1	2747	479		
	Control 10 + Estella_2	2	12999	332 and 487	4'4	**4n**
	Control 11	1	20260	600		
	Estella_3	1	10093	788		
	Control 11 + Estella_3	2	8416 and 8360	589 and 819	4'17	**4n**
	C37_2	1	14388	802		
	Control 11 + Estella_4	2	7825 and 9697	606 and 858	4'24	**4n**
	Control 12	1	5453	560		
	Estella_5	1	8147	851		
	Control 12 + Estella_5	2	14072 and 12797	570 and 817	4'3	**4n**

[a] individual that was also used for the genetic analysis

[b] the peak with a lower fluorescence value corresponds to sperm

[c] decrease in the florescence values of some samples due to a modification of the cytometer parameters

Table S4. List of the different TMED9 alleles obtained in this study. The individuals showing each allele and its corresponding GenBank accession number are indicated.

Allele	Individuals	GenBank accession number
T-1	Bosque1S	MK385658
T-2	Bosque1S	MK385769
T-3	Bosque1S, Bosque2S, Bosque3S	MK385790
T-4	Bosque3S	MK385801
T-5	Bosque2S	MK385812
T-6	Bosque1S	MK385823
T-7	Bosque3S	MK385834
T-8	Bosque3S	MK385845
T-9	Bosque2S	MK385856
T-10	Bosque2S	MK385659
T-11	Bosque2S	MK385670
T-12	Bosque1S	MK385681
T-13	Bosque1S	MK385692
T-14	Bosque2S	MK385703
T-15	Bosque3S	MK385714
T-16	Bosque3S	MK385725
T-17	Trelles1A, Trelles2	MK385736
T-18	Trelles1A, Trelles2, Trelles3S	MK385747
T-19	Trelles2	MK385758
T-20	Trelles3S	MK385770
T-21	Trelles3S	MK385781
T-22	Truchas1A, Truchas2	MK385782
T-23	Truchas1A	MK385783
T-24	SantaFe1A, SantaFe2A, SantaFe3A, Artavia1S	MK385784
T-25	Trelles1A	MK385785
T-26	Estella1A	MK385786
T-27	Estella2A	MK385787
T-28	Estella2A	MK385788
T-29	Estella2A	MK385789
T-30	Estella2A	MK385791
T-31	Truchas2	MK385792
T-32	Truchas2	MK385793
T-33	Artavia1S	MK385794
T-34	Artavia1S, Artavia2S, Artavia3S, Estella1A, Estella3	MK385795
T-35	Artavia2S	MK385796
T-36	Artavia2S	MK385797
T-37	Artavia3S	MK385798
T-38	Artavia3S	MK385799
T-39	Artavia3S	MK385800
T-40	Estella2A	MK385802
T-41	Estella3	MK385803
T-42	Estella3	MK385804
T-43	Artavia2S	MK385805
T-44	Artavia2S	MK385806
T-45	Estella1A	MK385807
T-46	Artavia3S	MK385808
T-47	Estella1A	MK385809
T-48	Estella3	MK385810
T-49	Artavia3S	MK385811
T-50	SantaFe1A	MK385813
T-51	SantaFe3A	MK385814
T-52	Artavia1S	MK385815
T-53	Truchas2	MK385816
T-54	Estella1A	MK385817

T-55	SantaFe1A	MK385818
T-56	SantaFe1A	MK385819
T-57	TrellesS2	MK385820
T-58	SantaFe1A	MK385821
T-59	Trelles3S	MK385822
T-60	Truchas2	MK385824
T-61	Truchas1A, Truchas3	MK385825
T-62	Truchas1A	MK385826
T-63	Trelles1A, Trelles3S	MK385827
T-64	Truchas2	MK385828
T-65	Truchas1A	MK385829
T-66	Truchas1A	MK385830
T-67	Truchas3	MK385831
T-68	Trelles1A, Trelles3S	MK385832
T-69	Trelles3S	MK385833
T-70	Trelles3S	MK385835
T-71	Trelles3S	MK385836
T-72	Trelles3S	MK385837
T-73	TrellesS2	MK385838
T-74	Truchas2	MK385839
T-75	Trelles1A	MK385840
T-76	Hortas1A	MK385841
T-77	Trelles2	MK385842
T-78	Trelles2	MK385843
T-79	Hortas1A	MK385844
T-80	Hortas1A	MK385846
T-81	Artavia1S, Estella1A, Estella2A	MK385847
T-82	Estella1A	MK385848
T-83	Estella1A	MK385849
T-84	Estella1A	MK385850
T-85	Estella3	MK385851
T-86	Estella3	MK385852
T-87	Estella3	MK385853
T-88	Artavia1S	MK385854
T-89	Estella2A	MK385855
T-90	Artavia1S	MK385857
T-91	Hortas1A	MK385858
T-92	Hortas5S	MK385859
T-93	Alte1	MK385860
T-94	Alte1, Alte2S	MK385861
T-95	Alte1	MK385862
T-96	Alte3S	MK385863
T-97	Alte3S	MK385864
T-98	Alte3S	MK385865
T-99	Alte3S	MK385866
T-100	Alte2S	MK385660
T-101	Alte2S	MK385661
T-102	Alte3S	MK385662
T-103	Alte2S	MK385663
T-104	Alte3S	MK385664
T-105	Alte2S	MK385665
T-106	Alte2S	MK385666
T-107	Estella2A	MK385667
T-108	Alte1	MK385668
T-109	Estella2A	MK385669
T-110	Hortas1A	MK385671
T-111	Hortas1A, SantaFe1A, SantaFe2A, SantaFe3A, Truchas1A, Truchas3	MK385672

T-112	Truchas3	MK385673
T-113	Truchas3	MK385674
T-114	Truchas3	MK385675
T-115	Truchas2	MK385676
T-116	SantaFe2A	MK385677
T-117	Hortas1A	MK385678
T-118	SantaFe2A	MK385679
T-119	SantaFe3A	MK385680
T-120	Truchas2	MK385682
T-121	SantaFe1A	MK385683
T-122	Trelles1A	MK385684
T-123	Trelles1A	MK385685
T-124	Truchas2	MK385686
T-125	Trelles2	MK385687
T-126	Trelles2	MK385688
T-127	Trelles2	MK385689
T-128	Hortas2A, Hortas5S	MK385690
T-129	Trelles3S	MK385691
T-130	Hortas5S	MK385693
T-131	Truchas3	MK385694
T-132	SantaFe1A, SantaFe2A, SantaFe3A, Truchas1A	MK385695
T-133	Hortas2A	MK385696
T-134	Truchas2	MK385697
T-135	SantaFe1A	MK385698
T-136	Truchas2	MK385699
T-137	Trelles3S	MK385700
T-138	SantaFe1A	MK385701
T-139	Trelles2	MK385702
T-140	SantaFe1A	MK385704
T-141	Hortas1A	MK385705
T-142	SantaFe3A	MK385706
T-143	SantaFe2A	MK385707
T-144	SantaFe1A	MK385708
T-145	SantaFe1A	MK385709
T-146	SantaFe1A	MK385710
T-147	SantaFe1A	MK385711
T-148	SantaFe1A	MK385712
T-149	SantaFe2A	MK385713
T-150	Hortas2A, Hortas3S	MK385715
T-151	Hortas1A	MK385716
T-152	Hortas3S	MK385717
T-153	Hortas3S	MK385718
T-154	Hortas2A	MK385719
T-155	Hortas2A	MK385720
T-156	Hortas5S	MK385721
T-157	Hortas5S	MK385722
T-158	Hortas2A	MK385723
T-159	Hortas2A	MK385724
T-160	Hortas3S	MK385726
T-161	Hortas3S, Hortas4S, Hortas5S	MK385727
T-162	Hortas3S	MK385728
T-163	Hortas4S	MK385729
T-164	Hortas5S	MK385730
T-165	Hortas4S	MK385731
T-166	Hortas3S	MK385732
T-167	Hortas4S	MK385733
T-168	Hortas3S	MK385734
T-169	Hortas4S	MK385735

T-170	Hortas1A	MK385737
T-171	Hortas2A	MK385738
T-172	Hortas5S	MK385739
T-173	Trelles1A	MK385740
T-174	Hortas2A	MK385741
T-175	Soller1S	MK385742
T-176	Soller3S	MK385743
T-177	Soller3S	MK385744
T-178	Soller2S	MK385745
T-179	Soller1S	MK385746
T-180	Soller1S	MK385748
T-181	Soller2S	MK385749
T-182	Soller1S	MK385750
T-183	Soller1S	MK385751
T-184	Soller3S	MK385752
T-185	Soller2S	MK385753
T-186	Soller2S	MK385754
T-187	Soller2S	MK385755
T-188	Soller3S	MK385756
T-189	Soller3S	MK385757
T-190	Soller3S	MK385759
T-191	Soller3S	MK385760
T-192	Soller2S	MK385761
T-193	Soller1S	MK385762
T-194	Soller3S	MK385763
T-195	Soller1S	MK385764
T-196	Soller1S	MK385765
T-197	Calobra1S, Calobra2S, Calobra3S,	MK385766
T-198	Calobra3S	MK385767
T-199	Calobra3S	MK385768
T-200	Calobra3S	MK385771
T-201	Calobra1S	MK385772
T-202	Calobra3S	MK385773
T-203	Calobra3S	MK385774
T-204	Calobra3S	MK385775
T-205	Calobra2S	MK385776
T-206	Calobra2S	MK385777
T-207	Calobra2S	MK385778
T-208	Calobra2S	MK385779
T-209	Calobra2S	MK385780
Outgroup sequences		
Species	Code	GenBank accession number
Dugesia hepta	MR1960	MK385867
	MR1962	MK385868
Dugesia benazzii	MR2191	MK385869
	MR2192	MK385870

Table S5. List of Cox1 haplotypes, with the individuals showing each haplotype and its corresponding GenBank accession number.

Haplotype	Individuals	GenBank accession number
C-1	Bosque1S, Bosque2S, Bosque3S	MK385871
C-2	Bosque2S	MK385872
C-3	Bosque1S	MK385873
C-4	Bosque3S	MK385874
C-5	Bosque3S	MK385875
C-6	Trelles2	MK385876
C-7	SantaFe1A, SantaFe2A, SantaFe3A, Trelles1A, Trelles2, Trelles3S, Hortas3S, Hortas4S, Hortas1A, Hortas5S, Truchas2, Truchas1A	MK385877
C-8	Trelles2	MK385878
C-9	Trelles1A, Trelles2	MK385879
C-10	Truchas2	MK385880
C-11	Trelles1A	MK385881
C-12	Hortas4S, SantaFe1A	MK385882
C-13	SantaFe3A	MK385883
C-14	Truchas1A	MK385884
C-15	SantaFe3A	MK385885
C-16	SantaFe1A	MK385886
C-17	Trelles3S	MK385887
C-18	Trelles3S	MK385888
C-19	Hortas4S	MK385889
C-20	Trelles2	MK385890
C-21	Trelles3S	MK385891
C-22	Trelles2	MK385892
C-23	Trelles1A, Trelles3S	MK385893
C-24	Trelles2	MK385894
C-25	Truchas2, Truchas1A, Truchas3	MK385895
C-26	Truchas1A	MK385896
C-27	Truchas 1A	MK385897
C-28	Truchas1A	MK385898
C-29	Truchas2	MK385899
C-30	Truchas2, Truchas3	MK385900
C-31	Truchas2	MK385901
C-32	Hortas2A	MK385902
C-33	Hortas2A, Hortas4S, Hortas5S	MK385903
C-34	Hortas2A	MK385904
C-35	Truchas2	MK385905
C-36	Alte1, Alte2S, Alte3S	MK385906
C-37	Alte3S	MK385907
C-38	Alte1	MK385908
C-39	Alte2S	MK385909
C-40	Truchas3	MK385910
C-41	Hortas5S	MK385911
C-42	Artavia1S, Artavia2S, Artavia3S, Estella1A, Estella2A, Estella3	MK385912
C-43	Artavia3S	MK385913
C-44	Soller1S	MK385914
C-45	Soller2S, Soller3S	MK385915
C-46	Soller3S	MK385916
C-47	Soller3S	MK385917
C-48	Soller3S	MK385918
C-49	Soller2S	MK385919
C-50	Calobra1S, Calobra3S, Calobra2S	MK385920
C-51	Calobra1S	MK385921

C-52	Calobra2S	MK385922
Outgroup sequences		
Species	Code	GenBank accession number
Dugesia hepta	MR1960	MK385923
	MR1962	MK385924
Dugesia benazzii	MR2191	MK385925
	MR2192	MK385926

TableS6. Genetic diversity and proportion of synonymous and nonsynonymous sites at intraindividual level for the two molecular markers analyzed in the present study

	**TMED9**					**Cox1**				
	$\boldsymbol{H_D}$	$\boldsymbol{\pi}$	Ks	Ka	Ω	$\boldsymbol{H_D}$	$\boldsymbol{\pi}$	Ks	Ka	Ω
**Sexual**										
Calobra1S	0.1333	0.0003	0	0		0.1429	0.0004	0.0009	0.0003	0.3125
Calobra2S	0.5714	0.0014	0.0063	0.0025	0.4031	0.25	0.0008	0	0.0010	
Calobra3S	0.6571	0.0024	0	0.0009		0		0	0	
Soller1S	0.8571	0.0050	0.0186	0.0018	0.0978					
Soller2S	0.641	0.0019	0	0.0020		0.0952	0.0024	0.0091	0.0004	0.0430
Soller3S	0.8476	0.0040	0.0091	0.0008	0.0926	0.3714	0.0021	0.0061	0.0008	0.1342
Bosque1S	0.5714	0.0010	0	0		0.1667	0.0005	0.0021	0	0
Bosque2S	0.5714	0.0016	0	0.0008		0.1667	0.0005	0	0.0007	
Bosque3S	0.5714	0.0018	0	0.0034		0.2747	0.0029	0.0113	0.0003	0.0266
Alte1	0.4231	0.0012	0.0073	0.0010	0.1349	0.1818	0.0008	0.0011	0.0007	0.6508
Alte2S	0.7802	0.0018	0	0.0018		0.2222	0.0010	0.0014	0.0009	0.6508
Alte3S	0.6818	0.0016	0.0040	0	0	0.1538	0.0005	0.0010	0.0003	0.3175
Artavia1S	0.8727	0.0071	0.0043	0.0023	0.5410	0	0	0	0	
Artavia2S	0.5385	0.0015	0	0		0	0	0	0	
Artavia3S	0.5714	0.0014	0.0031	0.0017	0.5450	0.1429	0.0007	0.0009	0.0006	0.6406
Σ=15	x̅=0.6193	x̅=0.0023	x̅=0.0035	x̅=0.0013	x̅=0.2592	x̅=0.1446	x̅=0.0008	x̅=0.0023	x̅=0.0004	x̅=0.3084
**Facultative**										
Hortas1A	0.9143	0.0137	0.0401	0	0	0	0	0	0	
Hortas2A	0.8857	0.0074	0.0126	0.0017	0.1343	0.6	0.0021	0.0042	0.0014	0.3254
HHortas3S	0.8485	0.0023	0.0079	0.0011	0.1327	0	0	0	0	
Hortas4S	0.4762	0.0014	0	0.0017		0.7143	0.0088	0.0326	0.0017	0.0529
Hortas5S	0.8571	0.0088	0.0188	0.0009	0.0487	0.4725	0.0097	0.0400	0.0007	0.0181
Trelles1A	0.9451	0.0119	0.0327	0	0	0.7121	0.0125	0.0495	0.0017	0.0339
Trelles2	0.9341	0.0144	0.0433	0.0018	0.0416	0.8333	0.0108	0.0402	0.0020	0.0496
Trelles3S	0.9619	0.0109	0.0184	0.0026	0.1408	0.5385	0.0077	0.0297	0.0012	0.0403
Σ=8	x̅=0.8529	x̅=0.0089	x̅=0.0184	x̅=0.0026	x̅=0.1408	x̅=0.4838	x̅=0.0065	x̅=0.0297	x̅=0.0012	x̅=0.0403
**Fissiparous**										
SantaFe1A	0.918	0.0125	0.0250	0.0005	0.0183	0.1775	0.0004	0	0.0006	
SantaFe2A	0.8846	0.0117	0.0247	0.0010	0.0392	0	0	0	0	
SantaFe3A	0.9286	0.0115	0.0207	0	0	0.3182	0.0010	0	0.0007	0.3254
Truchas1A	0.9011	0.0105	0.0356	0.0018	0.0513	0.6476	0.0104	0.0311	0.0042	0.1337
Truchas2	1	0.0146	0.0369	0.0043	0.1157	0.7308	0.0109	0.0325	0.0043	0.1310
Truchas3	0.8242	0.0080	0.0207	0.0018	0.0882	0.2949	0.0060	0.0197	0.0021	0.1046
Estella1A	0.9394	0.0091	0.0155	0.0032	0.2043	0	0	0	0	
Estella2A	0.8718	0.0076	0.0073	0.0010	0.1351	0	0	0	0	
Estella3	0.8762	0.0083	0.0127	0.0025	0.2003	0	0	0	0	

Σ=9	$\bar{x}$=0.9049	$\bar{x}$=0.0104	$\bar{x}$=0.0221	$\bar{x}$=0.0018	$\bar{x}$=0.0947	$\bar{x}$=0.241	$\bar{x}$=0.0032	$\bar{x}$=0.0095	$\bar{x}$=0.0013	$\bar{x}$=0.1737

H_D: haplotype diversity; π: nucleotide diversity; Ks: number of synonymous mutations / number of synonymous sites; Ka: number of nonsynonymous mutations / number of nonsynonymous sites; Ω: Ka/Ks

Table S7. Results of the statistical tests used to compare the intraindividual mean levels of genetic diversity and types of mutations between the different reproductive strategies.

Genetic parameter	Gene	Statistical test	p-value	p-value (Sexual vs Facultative)	p-value (Sexual vs Fissiparous)	p-value (Facultative vs Fissiparous)
H_D	Tmed9	H = 16.54	0.0003***	0.0079**	0.0007***	1
	Cox1	F = 5.696	0.0082**	0.0075**	0.6245	0.0649
π	Tmed9	F = 25.31	0.0000004***	0.0002***	0.0001***	0.477
	Cox1	F =6.663	0.0042**	0.0038**	0.3129	0.1159
Ks	Tmed9	F = 13.8	0.00006***	0.001**	0.0008***	0.9957
	Cox1	F = 8.151	0.0016**	0.0015**	0.4193	0.0331*
Ka	Tmed9	F^a = 0.5634	0.5801			
	Cox1	H= 2.869	0.2233			
Ω	Tmed9	F = 2.093	0.167			
	Cox1	H = 2.633	0.274			

H_D: haplotype diversity; π: nucleotide diversity; Ks: intraindividual proportion of synonymous mutations; Ka: intraindividual proportion of nonsynonymous mutations; Ω: ratio Ka/Ks

H: statistic of the Kruskal-Wallis test; F: statistic of the One-Way Anova test; F^a = statistic of the Welch test

(*p = 0.01-0.05; ** p = 0.001-0.01; *** p <0.001)
