# Supplementary material for: Outstanding intraindividual genetic diversity in fissiparous planarians (Dugesia, Platyhelminthes) with facultative sex
Source: BMC Evol Biol. 2019 Jun 20;19:130. doi: 10.1186/s12862-019-1440-1 (PMC6587288; doi:10.1186/s12862-019-1440-1)
Supplement: Supplementary file 2 — Figure S1. Workflow of the ploidy level estimation using flow cytometry. 1) Cutting the animal in two pieces, 2) cell maceration, 3) DNA staining, 4) fluorescence measurement with a Gallios flow cytometer and 5) unknown-ploidy estimation by comparison with control. Figure S2. Approach used in this study for the correction of the putative artifactual mutations due to polymerase mistakes. In parentheses is indicated the frequency of each haplotype within the individual before and after the correction. Figure S3. Bayesian inference tree of all the alleles of the nuclear gene TMED9. The colors of the terminal branches indicate to which individual each allele belongs. The color of the outer circle indicates the reproductive strategy of each individual. Numbers at the nodes indicate the support values for the Bayesian inference (posterior probability)/the maximum likelihood (bootstrap). Bootstrap values correspond to the maximum likelihood analysis conducted with rapid bootstrap. Support values lower than 0.8 (posterior probability) and 75% (bootstrap) are represented with a -. Scale bar indicates the number of substitutions per site. Figure S4. Bayesian inference tree of all the haplotypes of the mitochondrial gene Cox1. Haplotypes of the same individual are pictured with the same color. Numbers in white indicate the different clades within the Mixed clade. Numbers at the nodes indicate the support values for the Bayesian inference (posterior probability)/the maximum likelihood (bootstrap). Bootstrap values correspond to the maximum likelihood analysis conducted with rapid bootstrap. Support values lower than 0.8 (posterior probability) and 75% (bootstrap) are represented with a -. Scale bar indicates the number of substitutions per site. (PDF 878 kb) [file 12862_2019_1440_MOESM2_ESM.pdf]

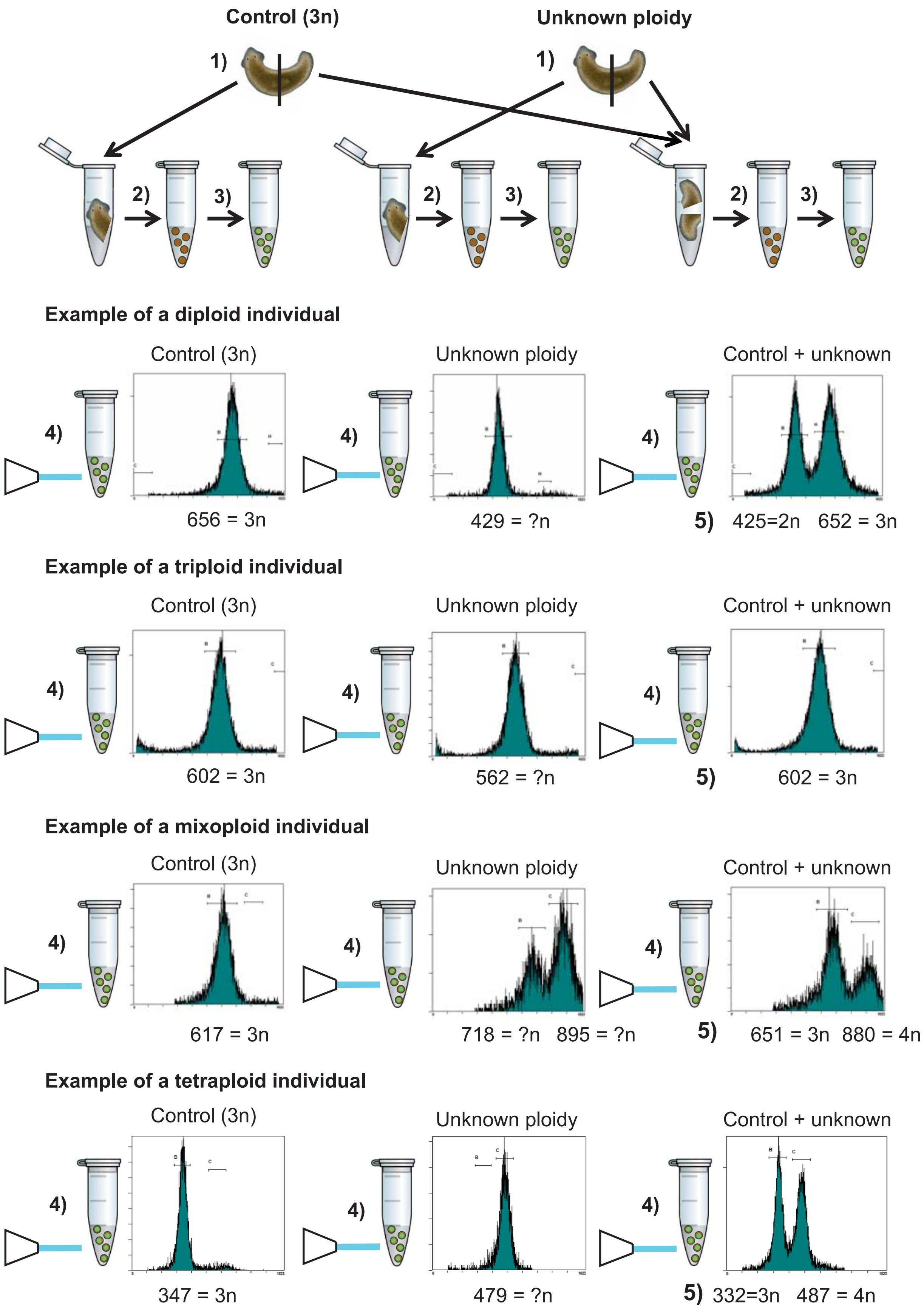

Figure S1. Workflow of the ploidy level estimation using flow cytometry. 1) Cutting the animal in two pieces, 2) cell maceration, 3) DNA staining, 4) fluorescence mesurement of each sample using a Gallios Flow Cytometer and 5) ploidy estimation of the unknown sample by comparison with the control.

Original sequence dataset for one individual

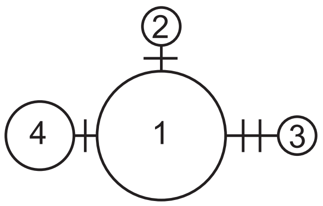

Seq 1: ATCGCATTAC (10)
Seq 2: ATCGCTTTAC (1)
Seq 3: ATGGCATAAC (1)
Seq 4: AACGCATTAC (3)

Dataset after the correction of putative artifact mutations

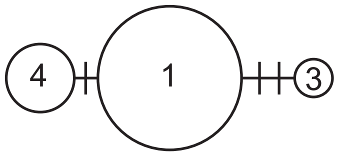

Seq 1: ATCGCATTAC (11)
Seq 3: ATGGCATAAC (1)
Seq 4: AACGCATTAC (3)

Figure S2. Approach used in this study for the correction of the putative artifactual mutations due to polymerase mistakes. In parentheses is indicated the frequency of each haplotype within the individual before and after the correction.

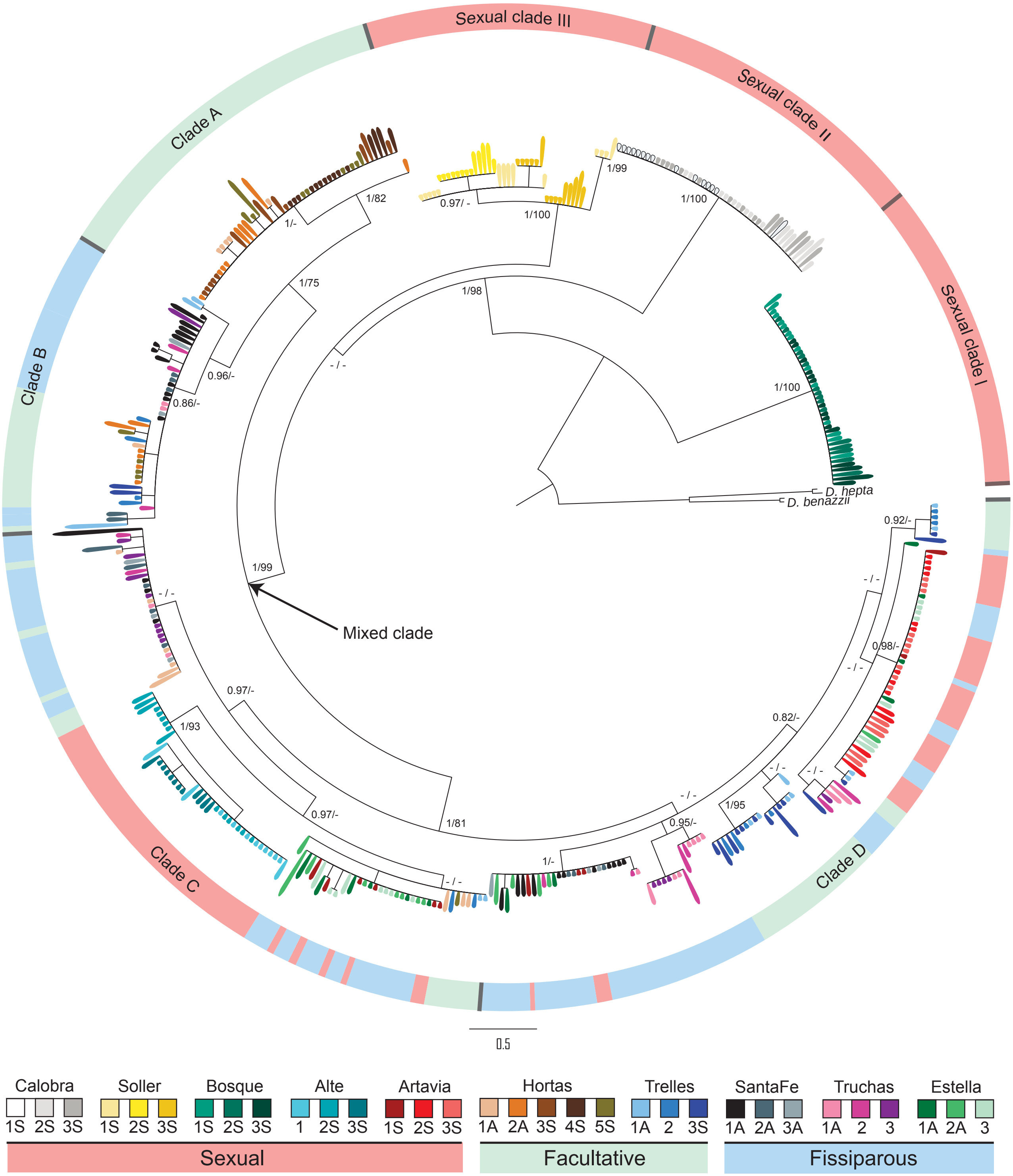

Figure S3. Bayesian inference tree of all the alleles of the nuclear gene TMED9. The colors of the terminal branches indicate to which individual each allele belongs. The color of the outter circle indicates the reproductive strategy of each individual. Numbers at the nodes indicate the support values for the Bayesian inference (posterior probability) and the maximum likelihood (bootstrap). Bootstrap values correspond to the maximum likelihood analysis conducted with rapid bootstrap. Support values lower than 0.8 (posterior probability) and 75% (bootstrap) are represented with a -. Scale bar indicates the number of substitutions per site.

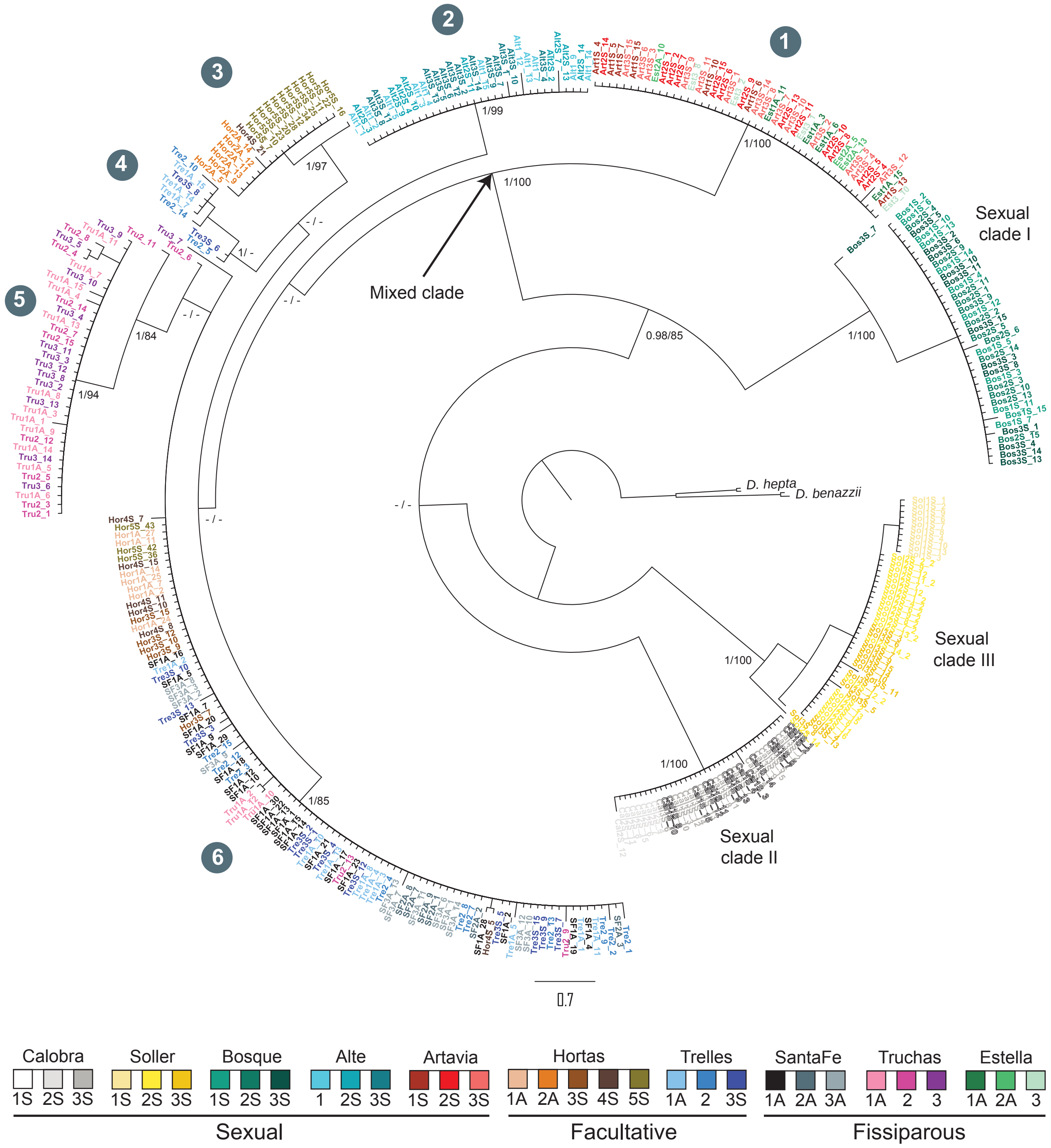

Figure S4. Bayesian inference tree of all the haplotypes of the mitochondrial gene Cox1. Haplotypes of the same individual are pictured with the same color. Numbers in white indicate the different clades within the Mixed clade. Numbers at the nodes indicate the support values for the Bayesian inference (posterior probability)/the maximum likelihood (bootstrap). Bootstrap values correspond to the maximum likelihood analysis conducted with rapid bootstrap. Support values lower than 0.8 (posterior probability) and 75% (bootstrap) are represented with a -. Scale bar indicates the number of substitutions per site.
